# Molecular Epidemiology of Underreported Emerging Zoonotic Pathogen *Streptococcus suis* in Europe

**DOI:** 10.3201/eid3003.230348

**Published:** 2024-03

**Authors:** Jaime Brizuela, Thomas J. Roodsant, Qureisha Hasnoe, Boas C.L. van der Putten, Jana Kozakova, Hans-Christian Slotved, Mark van der Linden, Ilse G.A. de Beer-Schuurman, Ewa Sadowy, Juan Antonio Sáez-Nieto, Victoria J. Chalker, Kees C.H. van der Ark, Constance Schultsz

**Affiliations:** Amsterdam University Medical Center, University of Amsterdam, Amsterdam, the Netherlands (J. Brizuela, T.J. Roodsant, Q. Hasnoe, B.C.L. van der Putten, I.G.A de Beer-Schuurman, K.C.H. van der Ark, C. Schultsz);; National Institute for Public Health, Prague, Czech Republic (J. Kozakova);; Statens Serum Institut, Copenhagen, Denmark (H.-C. Slotved);; University Hospital RWTH Aachen, Aachen, Germany (M. van der Linden);; National Medicines Institute, Warsaw, Poland (E. Sadowy);; Carlos III Health Institute, Madrid, Spain (J.A. Sáez-Nieto);; UK Health Security Agency, London, UK (V.J. Chalker).

**Keywords:** Streptococcus suis, zoonoses, bacteria, pathogens, porcine diseases, pork, pigs, swine, whole genome sequencing, molecular epidemiology, systematic review, Europe

## Abstract

*Streptococcus suis*, a zoonotic bacterial pathogen circulated through swine, can cause severe infections in humans. Because human *S. suis* infections are not notifiable in most countries, incidence is underestimated. We aimed to increase insight into the molecular epidemiology of human *S. suis* infections in Europe. To procure data, we surveyed 7 reference laboratories and performed a systematic review of the scientific literature. We identified 236 cases of human *S. suis* infection from those sources and an additional 87 by scanning gray literature. We performed whole-genome sequencing to type 46 zoonotic *S. suis* isolates and combined them with 28 publicly available genomes in a core-genome phylogeny. Clonal complex (CC) 1 isolates accounted for 87% of typed human infections; CC20, CC25, CC87, and CC94 also caused infections. Emergence of diverse zoonotic clades and notable severity of illness in humans support classifying *S. suis* infection as a notifiable condition.

*Streptococcus suis* is an opportunistic bacterial porcine pathogen that can cause severe disease in humans, most commonly meningitis and sepsis (*1*). Human *S. suis* infections occur both through direct contact with infected pigs and consumption of undercooked contaminated pork (*2*). Human *S. suis* infections have become endemic in Thailand and Vietnam, driven by consumption of traditional raw pork dishes (*1*), and *S. suis* has caused multiple outbreaks in humans with high levels of illness and death in China and Thailand (*3*). In Europe, *S. suis* infections are considered an occupational hazard, mainly occurring among persons with skin lesions working closely with pigs or pork products (*1*). Human infections in Europe account for ≈10% of the global prevalence, but incidence in Europe is likely underestimated because *S. suis* infections are not a notifiable disease (*4*). Togo, Madagascar, Chile, and Indonesia have recently reported zoonotic *S. suis* infections, meaning all continents except Antarctica have now reported human infections (*1*,*5*–*8*). 

*S. suis* is classified into 29 distinct serotypes based on its capsular polysaccharide, as well as 27 novel serotypes based on novel capsular polysaccharide loci. In addition, sporadic infections caused by serotypes 4, 5, 7, 9, 16, 21, 24, and 31 have been reported (*3*,*9*–*11*). *S. suis* genotypes are classified on the basis of sequence types (STs) determined through multilocus sequence typing (MLST), which are grouped into clonal complexes (CCs) (*12*). CC1 with a serotype 2 capsule is the main lineage causing human infections and has expanded worldwide (*3*). Emerging zoonotic lineages, such as CC20, which emerged from CC16 in the Netherlands after acquiring a serotype 2 capsule, have also been described (*13*). 

We aimed to increase insight into the epidemiology of human *S. suis* infections in Europe and to assess the bacterial population structure and diversity of zoonotic *S. suis* clades (*1*). We assessed the frequency of human *S. suis* infections in Europe through a survey of reference laboratories in top pig-rearing countries in Europe, performed a systematic literature review and explored the gray literature (social media, news accounts, and government reports). In addition, we reconstructed a representative phylogeny of zoonotic *S. suis* isolates in Europe. 

This study was not reviewed by an ethics review board, because it was based on anonymized surveillance data. In accordance with Dutch law, approval from a medical ethics committee was not deemed necessary because case-patients were not subject to any actions or rules of conduct. We did not obtain informed consent because our data collection processes were exempted under exceptions formulated in the Dutch Implementation of the European General Data Protection Regulation Act (2016/679). 

## Methods 

### Survey 

We contacted national reference laboratories in 10 countries in Europe (Czech Republic, Denmark, France, Germany, Hungary, Italy, the Netherlands, Poland, Spain, and the United Kingdom) that included *S. suis* infections within their scope. We asked those laboratories to retrospectively collect data on cases of human *S. suis* infection during 1990–2018 because most human *S. suis* infections have been reported since 1990. We asked participating laboratories to complete a questionnaire collecting patient metadata and bacterial typing and metadata. Anonymized patient metadata were age, sex, clinical signs, and occupation. Bacterial typing encompassed serotype, sequence type (ST), and available whole-genome sequences. Bacterial metadata were date of isolation, source of isolation, and method of identification. In addition, we requested in the questionnaire that reference laboratories share their isolates for further genomic analysis (Appendix). 

### Systematic Review 

**Figure 1:**
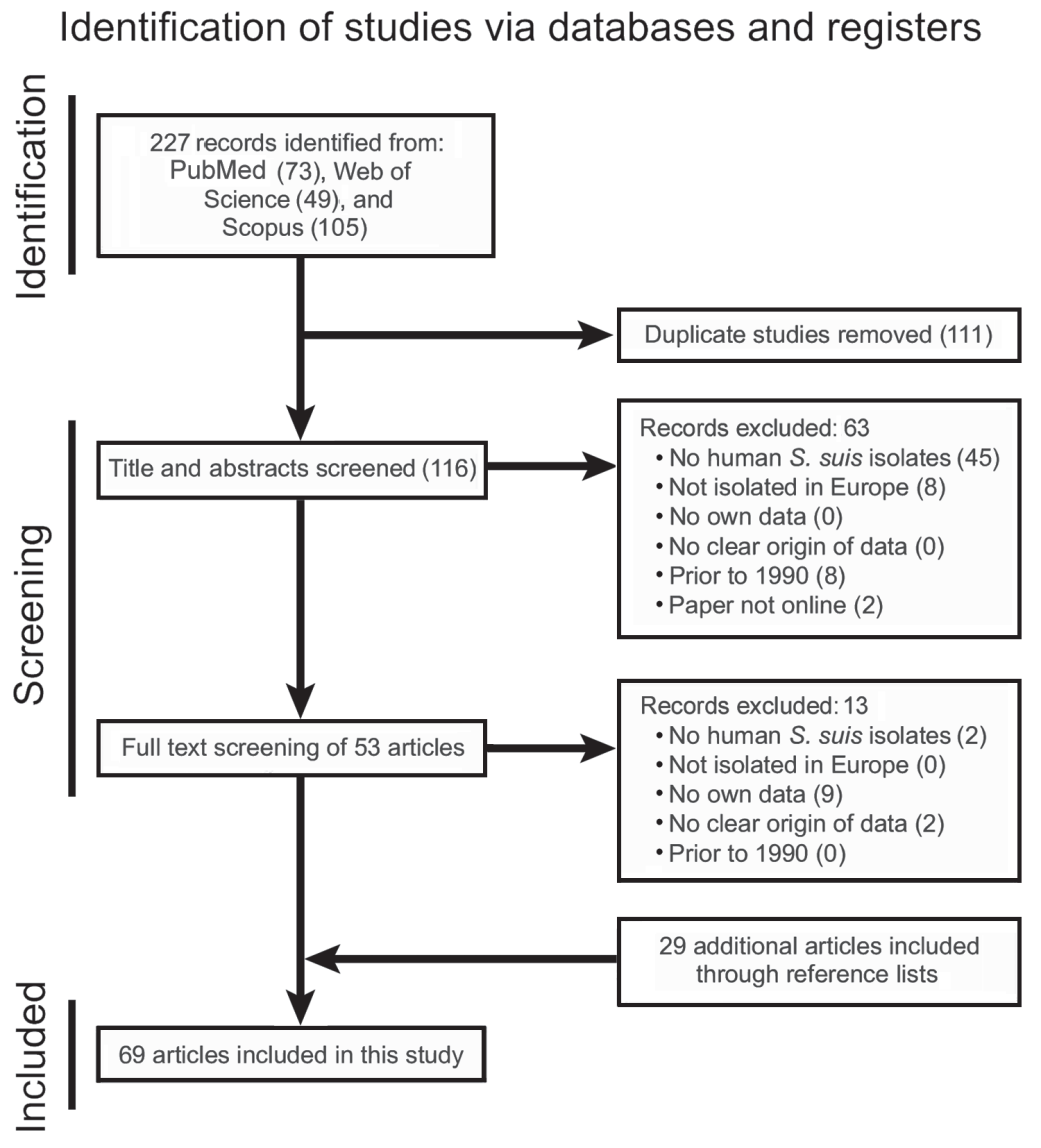
Preferred Reporting Items for Systematic Reviews and Meta-Analyses (PRISMA) search flowchart for systematic review of *Streptococcus suis* in Europe during 1990–2022.

We performed a systematic review according to PRISMA (Preferred Reporting Items for Systematic Reviews and Meta-Analyses) guidelines (*14*) to identify cases of human *S. suis* infections in Europe in articles published from 1990 (survey start date) through 2022. We screened PubMed, Web of Science, and Scopus for key terms—*S. suis,* human, and ≥1 country in Europe (as defined by the World Health Organization)—in the titles or abstracts of articles published before April 1, 2022 (Appendix). We removed duplicate references by using Zotero version 6.0.8 (https://www.zotero.org) and manual checking. We included studies containing data on human *S. suis* isolates or case reports describing human *S. suis* infections in Europe; we extracted patient and bacterial metadata for further analysis. We excluded studies that did not include data on zoonotic *S. suis* isolates or human infections, reported isolates not collected in Europe, did not publish original data, were published before 1990, or lacked information on the origin of isolates (Figure 1). To avoid duplication, we excluded from the systematic review isolates reported in both the survey and an article; in addition, if an isolate appeared in multiple articles, we included data only from the original article. 

### Grey Literature Search 

Because *S. suis* is not a notifiable disease, there are no guidelines for reporting such infections. To identify additional cases, we performed a broad scan of gray literature to capture cases of human *S. suis* infections in Europe not identified in the scientific literature or the survey. However, we distinguished cases we identified in the survey, literature review, and official reports from unreported cases (all other cases). We searched X (previously Twitter) and the Google news section using the terms *S. suis,* infection, and human in Dutch, English, French, German, Italian, Portuguese, and Spanish. To complement those data, we scanned ministry of health websites from France, Germany, Italy, the Netherlands, Portugal, Spain, and the United Kingdom for reports on zoonotic bacterial infections related to human *S. suis* infections. To avoid duplication, we compared metadata, when available, with isolate data from the survey and systematic review. 

### In Silico Typing and Phylogenetic Analysis 

We included 74 genomes for whole-genome sequencing (WGS) analysis, 67 from the survey (46 sequenced during this investigation and 21 previously sequenced) and 7 from the systematic review (Appendix Figure 1). We used MLST version 2.19.0 (https://github.com/tseemann/mlst) with the PubMLST database (https://pubmlst.org) to type the MLST profiles of the draft genomes. We submitted profiles for novel STs to PubMLST. We performed in silico serotyping by feeding processed Illumina reads into the *S. suis* serotyping pipeline (*15*). We reconstructed a core genome single-nucleotide polymorphism (SNP) phylogeny and using Panaroo version 1.3.0 (*16*) to reconstruct the pangenome and align the core genome. We calculated the number of constant sites in the core genome alignment with SNP-sites version 2.5.1 (*17*) using the flag “-C.” We reconstructed the maximum-likelihood (ML) phylogeny by running IQ-TREE version 2.0.3 (*18*) with 1,000 bootstraps and used the general time-reversible plus gamma model with the flag “-fconst” to include the constant sites from SNP-sites. We investigated the presence of 46 accessory genes previously found to be overrepresented in zoonotic isolates (human-pig prevalence ratio >2) using ABRicate (https://github.com/tseemann/abricate) with a custom database and a minimum protein identity and coverage of 80%. We visualized the resulting gene presence/absence matrix in Phandango (*19*,*20*). Raw Illumina sequences can be found in the National Center for Biotechnology Information Short Read Archive (BioProject PRJNA853715). Genome assemblies have been deposited in GenBank and are available under the same BioProject number (Appendix Table 7). 

## Results

### Geographic Distribution of Reported Human *S. suis* Infections across Europe, 1990–2022 

**Figure 2:**
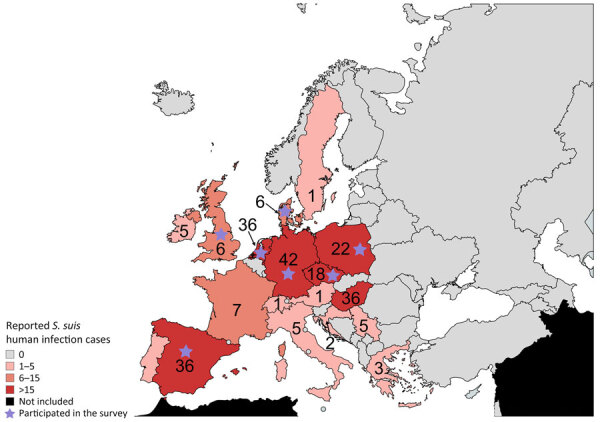
Reported cases of human *Streptococcus suis* infections across Europe during 1990–2022. We pooled reported cases collected in the survey study and systematic search study. The color of the countries represents the relative number of cases: the darker the tone, the higher the number of reported cases. Scale bar indicated substitutions per site. Purple stars indicate reference laboratory participating in the survey study within that country. Countries in black were not included in the study.

Of 10 reference laboratories invited to participate in the survey, 7 laboratories (Spain, Germany, Netherlands, Denmark, Czech Republic, Poland, and United Kingdom) responded and reported 107 unique cases of human *S. suis* infections (Appendix Table 2). In the systematic review, of 119 screened titles and abstracts, we selected 53 articles mentioning human *S. suis* infections in Europe for full-text reading. In addition, we included 29 studies identified by screening reference lists (Figure 1). In total, we extracted data from 129 cases of human *S. suis* infections reported in 69 research articles (Figure 1; Appendix Table 3). Combining both sources, we identified 236 unique cases of human *S. suis* infections across Europe during 1990–2022. Germany, Spain, and the Netherlands, the top pig-rearing countries in Europe (*21*), reported 114/236 (48%) of the cases (Figure 2). Furthermore, 203/236 (86%) of the reported cases originated from just 8 countries (Germany, Spain, the Netherlands, Denmark, Hungary, France, Poland, and the Czech Republic), 6 of which participated in the survey study; sporadic cases reported from 8 additional countries in Europe completed the dataset. 

### Epidemiology of Human *S. suis* Infections in Europe 

Most patients were middle-aged men (Table 1). Of patients with a reported clinical syndrome, meningitis was the main clinical syndrome observed in both the survey (59/71 [83%]) and systematic review (59/86 [68%]), followed by sepsis, which affected 15/71 (21%) in the survey and 21/86 (24%) in the systematic review. Additional clinical signs and symptoms included hearing loss (n = 22), endocarditis (n = 6), and spondylodiscitis (n = 3); 11 patients died. Patient occupation was described as a potential risk factor in 19 cases in the survey and 72 cases in the systematic review (Table 1). Most infections, 78/92 (85%) in the survey and 43/48 (90%) in the systematic review, were caused by serotype 2 isolates, followed by serotype 14 isolates (Table 2). Most isolates (76/87 [78%] in the survey and 14/16 [88%] in the systematic review) belonged to zoonotic lineage CC1. In addition, 11/87 (13%) infections in the survey and 1 in the systematic review were caused by CC20 lineage isolates. 

**Table 1:** Patient data collected in survey and systematic review for study of molecular epidemiology of underreported emerging zoonotic pathogen *Streptococcus suis*, Europe*

Patient data	Survey, n = 107	Systematic review, n = 129
Demographic information		
Sex		
M	73	93
F	16	24
NA	18	12
Age		
Median (range), y	52 (0–79)	48 (22–85)
NA	26	54
Clinical symptoms		
Meningitis	59	59
Sepsis	15	21
Hearing loss	0	22
Endocarditis	2	6
Spondylodiscitis	0	3
Death	0	11
NA	36	43
Occupational risk†		
Described	19	72
No risk	0	2
NA	88	55
*Values are no. patients except as indicated. NA, not available. †Occupational risk: Any job involving close contact with pigs or pork products, including: farmer, butcher, abattoir worker, meat factory worker, hunter, livestock truck driver, or cook.		

**Table 2:** Bacterial isolate data collected in survey and systematic review for study of molecular epidemiology of underreported emerging zoonotic pathogen *Streptococcus suis*, Europe*

Isolate data	Survey, n = 107	Systematic review, n = 129
Serotype no.		
2	78	43
5	2	1
7	1	0
14	11	4
NA	15	81
Clonal complex no.		
1	68	14
20	11	1
25	4	0
87	3	1
94	1	0
NA	20	113
*NA, not available.		

Year of isolation was collected for only 44/129 (34%) isolates from cases in the systematic review (Appendix Table 3). Nonetheless, average number of cases per year in the systematic review and survey increased after 1999, from 2.7 during 1990–1999 to 5.7 during 2000–2009 and 5.0 during 2010–2019 (Appendix Figure 2). Moreover, we calculated crude estimates of *S. suis* incidence in the at-risk population in 6 (Czech Republic, Germany, Hungary, the Netherlands, Poland, and Spain) countries with >5 cases reported in the survey or literature review during 2005–2013. We defined the population at risk as the proportion of the agricultural census involved in pig specialized holdings with a 10% upper margin to account for butchers, hunters, slaughterhouse workers, lorry drivers, and meat factory workers. Incidence range in the at-risk population for those 6 countries during 2005–2013 averaged 0.161–4.945 cases/100,000 persons; Poland had the lowest incidence and the Netherlands the highest (Appendix Table 6). 

### Scan of Grey Literature

Because no centralized surveillance system exists for human *S. suis* infection and the disease is not notifiable in any country in Europe, the number of infections in Europe has likely been underestimated. We scanned gray literature in search of cases not identified through either the survey or systematic review. Public Health England (now the UK Health Security Agency) included human *S. suis* infections in their annual zoonosis official reports collected from the Veterinary Diagnostic Analysis database of the Animal and Plant Health Agency (*22*). During 1991–2017, those reports recorded 61 human *S. suis* infections in the United Kingdom, 10 times the number of cases identified from the survey and systematic review combined (6 cases). However, those 61 cases might overlap with cases from the survey and systematic review because neither metadata nor identification method were provided (Appendix Table 4). The Netherlands Reference Laboratory for Bacterial Meningitis surveyed 57 medical microbiology laboratories in the Netherlands during 2013 with the aim of identifying cases not reported to the reference laboratory and collected an additional 25 unique cases isolated during 1990–2011 (Appendix Table 5) (*23*). We also found 1 case of *S. suis* meningitis in a butcher in Spain that was reported through X (*24*). 

### Population Structure of Zoonotic *S. suis* in Europe 

**Figure 3:**
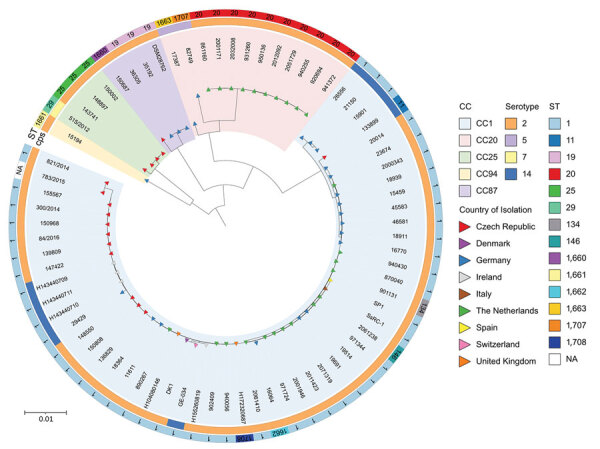
Genome population structure of zoonotic *Streptococcus suis* in Europe. The maximum-likelihood tree was reconstructed using IQ-TREE (18) with a core genome alignment produced with Panaroo (16). Color triangles at branch tips indicate country of collection; color rings indicate lineage (CC). The inner color ring indicates ST and is labeled accordingly. The outer color ring indicates the serotype as determined by the antigenic properties of the cps. We used iTOL (https://itol.embl.de) to visualize the tree. cps, capsular polysaccharide; NA, not available; ST, sequence type.

To study the population structure of zoonotic *S. suis* isolates in Europe, we reconstructed a core-genome SNP phylogeny of 74 strains from 10 different countries (Figure 3). We identified 5 novel STs, 1660, 1602, 1663, 1707, and 1708. Most strains were part of the major zoonotic clade CC1, which has spread across Europe; >1 strain from each country included in the phylogeny was CC1. Most of the CC1 strains had a serotype 2 capsule, and a small subset possessed the structurally similar serotype 14 capsule (*25*). We distinguished 2 subclades within CC1 in a genome-wide SNP phylogeny (Appendix Figure 3). The other zoonotic clades appeared to be more geographically restricted. For example, most of the CC20 strains were isolated in the Netherlands, where the lineage is thought to have emerged (*13*). Two additional CC20 strains were isolated in Germany, forming a serotype 5 outgroup to clonal CC20 serotype 2 strains from the Netherlands. All CC25 strains were recovered in the Czech Republic. The 3 ST25 serotype 2 strains had only 73–116 SNPs across their core genomes, whereas the ST29 strain differed from the ST25 strains by 4,353–4,416 SNPs and had a serotype 7 capsule. Strains from the CC87 clade were identified in Germany and the Czech Republic and possessed a serotype 2 capsule. The 3 strains from Germany were ST19 and highly similar (81–118 SNPs), whereas the strain from the Czech Republic had novel ST1660 and differed from the ST19 clade by 9,411–9,434 SNPs. 

### CC1 and CC20 Isolates and Genes Associated with Zoonotic Potential

**Figure 4:**
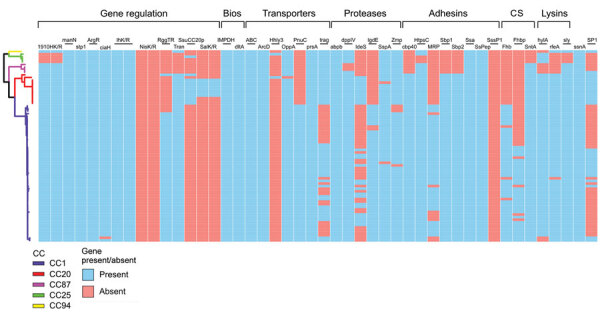
Presence/absence matrix of 46 genes putatively associated with zoonotic potential in study of zoonotic *Streptococcus suis* in Europe. The same phylogenetic tree presented in Figure 3 was used. Blue squares indicate presence of the gene while red squares indicate absence. The colored branches indicate CCs and follow the same pattern as in Figure 3 (blue, CC1; red, CC20; purple, CC87; yellow, CC94; green, CC25). We defined gene presence with 80% protein identity and coverage. We used Phandango (*19*) to visualize the tree. Bios, biosynthesis; CC, clonal complex; CS, complement system evasion.

Overall, strains from clades CC1 and CC20 had a higher number of accessory genes overrepresented in zoonotic isolates than did strains from lineages CC25, CC87, and CC94 (Figure 4). Most genes associated with zoonoses were present in >1 of the lineages; only the 2-component signal transduction system *nisK/R* and the fimbria-like adhesin *sssP1* genes were absent from the dataset (Figure 4). Of note, despite its role in adhesion and virulence being extensively studied, muramidase-related protein (*mrp*) was absent from the CC20 clade (*26*). Factor H binding protein (*fhb*), associated with binding factor H and increased translocation across the blood/brain barrier (*27*), was present only in CC1 strains. Differences could be observed within CC1 sublineages; 1 subclade had an additional factor H binding protein (*fhbp*)*.* Last, suilysin (*sly*), a pore-forming hemolysin with a clear role in pathogenesis (*20*), was present in all clades except CC25, which instead carried the hyaluronate lysin A (*hylA*), associated with reduced virulence (Figure 4) (*28*). 

## Discussion 

Despite having caused multiple outbreaks with high levels of illness and death in the past decade and reports of new zoonotic lineages arising on different continents, *S. suis* remains largely excluded from disease surveillance programs (*29*). Although neither carriage in healthy humans nor human-to-human transmission of *S. suis* have been reported to date, systematic surveillance is needed to follow the evolutionary trends of this pathogen in humans and pigs, the main reservoir from which zoonotic lineages emerge (*2*). 

Our study included some potential sources of bias. Differences in number of cases between countries should not be attributed only to the size of pig populations. Other factors, such as government policy and disease monitoring and reporting, could contribute to observed differences in reported human *S. suis* cases between countries. For instance, although France has one of the largest pig populations in Europe, only 7 human cases have been reported. In contrast, although they have smaller pig populations, the Czech Republic reported 18 and Poland 22 cases (Figure 2) (*30*). The distribution of clinical symptoms aligns with previous regional and global estimates (*1*). However, clinical data gathered in the survey were potentially biased toward reporting meningitis because several of the surveyed laboratories are reference laboratories for bacterial meningitis (Table 1) (*1*). The systematic review yielded diverse article types (e.g., case reports, surveillance studies) and inconsistent quality of reported metadata. Often, year of isolation and bacterial typing was absent, making it difficult to establish meaningful time trends in the emergence of zoonotic *S. suis* in Europe. Finally, the time frames of the survey, 1990–2018, and systematic review, 1990–2022, were not identical. 

The serotype 2 capsule is linked with zoonotic *S. suis* infections, and most worldwide *S. suis* cases are caused by serotype 2 (*4*). While investigating the emergence of the zoonotic clade CC20, 1 study (*13*) proposed that capsule-switching events leading to acquisition of a serotype 2 capsule may be necessary for pathogenic porcine strains to become zoonotic. We observed hints of capsule-switching events, with the CC20 strains from Germany carrying serotype 5 capsule instead of serotype 2, potentially representing an intermediate step in the emergence of zoonotic CC20 from CC16 (*13*). Furthermore, zoonotic strains from CC87 and CC94 lineages were serotype 2, whereas most porcine CC87 strains described in the literature carried a serotype 8 capsule; porcine CC94 strains displayed a wide range of capsules, serotypes 3, 7, and 23 being the most common (*31*). However, the low number of samples collected for CC25, CC87, and CC94 in our study and the fact that they were collected more than a decade ago make it difficult to conclude whether or not these CCs are emerging as zoonotic lineages or are geographically restricted (Appendix Tables 2, 3). 

The presence of genes associated with zoonotic potential varied across lineages. Differences in the accessory genome of the zoonotic *S. suis* population, with some well-studied virulence factors such as *sly*, *mrp*, and *fhb* missing from certain pathogenic clades, suggest that, although individual genes might contribute to virulence and zoonotic potential, those genes are not individually essential for *S. suis* to infect humans (*20*,*26*,*27*) (Figure 4). Moreover, simply because a gene is overrepresented in zoonotic isolates does not mean it plays an active role in zoonotic potential, and its role in zoonosis should be explored experimentally. For example, some genes, such as *zmp* and *sp1*, more common in human than porcine *S. suis* isolates, have been shown not to be critical for virulence (*32*,*33*), and others, such as *igdE* and *ideS*, only play a role in evading porcine, not human, immune response (*34*,*35*). 

Estimated cumulative prevalence of human *S. suis* infection is substantially higher in southeastern Asia than Europe and the epidemiology of human *S. suis* infections differs significantly between continents (*1*). In Europe, skin injuries and abrasions are thought to be the main point of entry for *S. suis* (*3*), whereas in countries in southeastern Asia with a tradition of raw pork product consumption, the intestinal tract is a notable entry point for infection (*2*,*36*). Differences in exposure routes have led to differences in epidemiology; multiple foodborne human *S. suis* outbreaks with high levels of illness and death have occurred in southeastern Asia in the past 2 decades (*36*,*37*). In Thailand, educational campaigns targeted toward at-risk populations have been shown to reduce incidence of human infections (*36*). Educational campaigns in Europe should be tailored to the different at-risk populations there. Our crude estimates of incidence of *S. suis* human infections in the population at risk for the Czech Republic, Germany, Hungary, the Netherlands, Poland, and Spain are comparable to the incidence of other pathogens causing similar infections in the general population (Appendix). Our estimated incidence in the population at risk for *S. suis*, range 0.161–4.945 cases/100,000 persons across the different countries, was generally higher (except in Poland) than population-wide incidence for *Neisseria meningitidis* (0.42–1.09) and lower than that of *S. pneumoniae* (1.52–14.86) reported by the European Centre for Disease Prevention and Control (*38*) (Appendix Table 6). 

Furthermore, we found evidence of underreporting in the Netherlands; 25 cases were not reported to the Netherlands Reference Laboratory for Bacterial Meningitis or described in published articles (*23*). The United Kingdom was the only country where human *S. suis* infections were included in official government reports. Those UK reports contained 10 times as many cases within the same timeframe than UK cases from the survey and systematic review combined (*22*) because the survey and systematic review did not capture many unpublished cases. This finding suggest that the number of cases collected in other countries through the survey might also be underestimated. We observed an increase in reported cases after 1999 (Appendix Figure 2); however, this increase could have been caused by heightened awareness after a severe outbreak in China in 2005 and by more precise bacterial identification techniques (*37*). Moreover, in Thailand, a country where *S. suis* is a notifiable disease, reported infections have increased in the past few years (*10*). 

In conclusion, despite not being a notifiable disease in Europe, novel zoonotic *S. suis* lineages, including multidrug-resistant lineages, have been detected recently both in Europe and worldwide (*13*,*29*). Moreover, our likely underestimated incidence estimates suggest that risk for *S. suis* infection for the at-risk population is greater than that of *N. meningitidis* and comparable to that of *S. pneumoniae* in the general population. Given the severity of the disease it causes, we propose making *S. suis* infections notifiable in Europe to improve surveillance of emerging zoonotic lineages and evolutionary trends and better detect potential human-to-human transmission. 

Appendix Additional information about study of human *Streptococcus suis* infections across Europe during 1990–2022.
