# Supplementary material for: Molecular Epidemiology of Underreported Emerging Zoonotic Pathogen Streptococcus suis in Europe
Source: Emerg Infect Dis. 2024 Mar;30(3):413–22. doi: 10.3201/eid3003.230348 (PMC10902550; doi:10.3201/eid3003.230348)
Supplement: Appendix — Additional information about study of human Streptococcus suis infections across Europe during 1990–2022. [file 23-0348-Techapp-s1.pdf]

Article DOI: https://doi.org/10.3201/eid3003.230348

EID cannot ensure accessibility for Appendix materials supplied by authors. Readers who have difficulty accessing Appendix content should contact the authors for assistance.

Molecular Epidemiology of Underreported Emerging Zoonotic Pathogen *Streptococcus suis*, Europe

Appendix

I. Questionnaire requesting patient and isolate data of *S. suis* infections.

Below are listed the questionnaire and supplied response options. Variables included region of coverage of the laboratory and population demographics (general information) as well as information on isolate and patient metadata. If predetermined response was not provided, responder was able to fill out the adequate response. If willing, laboratories were requested to send their isolates for future research.

1. General information
   a. What is the region covered by your reference laboratory?
   b. What is the population size for the above stated region?
   c. If known, what is the size of the population at risk* for the above stated region?
   d. If known, what is the size of the pig population for the above stated region?

   *The population at risk in Europe is defined as the population that includes all pig farmers, butchers, slaughterhouse workers, and hunters of wild boar.
2. Information on *Streptococcus suis*
   a. Is *S. suis* infection a notifiable disease in your country? (Y/N)
   b. What is the total number of *S. suis* cases notified to your laboratory from 1990 to date?
   c. If applicable, when was the first notified case of *S. suis* in your country? (dd/mm/yyyy)
3. Patient information

Below is the format used for requested information:

Patient information						
Date of birth	Sex	Clinical signs	Occupation	History of cuts and/or skin abrasions in the 14 days prior to infection	Residential area	Recent travel history

Variables including definitions and provided response options:

- Date of birth. Response format: dd-mm-yyyy.
- Sex. Response options: Male / Female.
- Clinical signs

  Definition: known clinical signs of patient (written on sample submission form)

  Response options: Meningitis/ Septicaemia/ Meningitis and septicaemia/ If other, specify.
- Cuts and/or abrasions in the 14 days prior to infection

  Definition: cuts and/or abrasions in the skin; described in the literature as a port of entry in population at risk. Response options: Yes/ No/ Unknown/ If other, specify.
- Occupation

  Definition: occupation of patient (if known).

  Response input: If available please enter the occupation of the patient.
- Residential area

  Definition: living area of patient, i.e. district, city and/or region (if known).

  Response input: If available please enter living area of patient, i.e. district, city and/or region.
- Recent travel history

  Definition: travel history of patient, i.e. country, city, region (if known).

  Response input: If known please enter country, city, region, etc. where patient has travelled to.

4. Isolate information

The format used to request isolate data:

	Isolate information							
	Isolate ID	Date of isolation	Isolated from	Isolate available	Identification method	Serotype	MLST sequence type	Whole genome sequenced
1								
2								
3								
4								
5								
6								
7								
8								
9								
10								

Variables including definitions and provided response options:

- Isolate ID

  Definition: ID or number assigned to an incoming patient sample, i.e. liquor, blood or other.

  Response input: variable according to reference laboratory-specific format.

- Date of isolation

  Definition: Date the sample arrived at the ref lab

  Response format: dd-mm-yyyy

- Isolated from

  Definition: source of harvest of isolate or sample from patient.

  Response options: CSF/ Blood / Other, namely

- Isolate available

  Definition: is the isolate stored in the ref lab and available to eventually send for follow-up.

  Response options: Yes/ No

- Identification method

  Definition: method via which bacterial typing was done.

  Response options: API Strep/ MALDI-TOF/ PCR, please specify target/ VITEK/ WGS/ If other, specify.

- Serotype

  Definition: known S. suis serotypes to date, i.e. 1, 1/2, 2, …, 34.

  Response options: Please enter numerical value; options: 1, ½, 2, …, 34/ Undetermined.

- Sequence type

  Definition: S. suis sequence type determined by MLST

  Response format: If determined, fill out value.

- Whole genome sequenced

  Definition: if whole genome sequencing has been performed

  Response options: Yes/ N

5. Follow-up

Would you be willing to ship *S. suis* isolates to the Dutch National Reference Laboratory for further analysis through whole genome sequencing? (Y/N)

II. Searches

Search Queries used in the Systematic review

PubMed Query

("Streptococcus suis" [MESH] OR "Streptococcus suis" [TIAB]) AND (human [MESH] OR human [TIAB]) AND ((Albania [MESH] OR Albania [TIAB]) OR (Andorra [MESH] OR Andorra [TIAB]) OR (Armenia [MESH] OR Armenia [TIAB]) OR (Austria [MESH] OR Austria [TIAB]) OR (Azerbaijan [MESH] OR Azerbaijan [TIAB]) OR (Belarus [MESH] OR Belarus [TIAB]) OR (Belgium [MESH] OR Belgium [TIAB]) OR ("Bosnia and Herzegovina" [MESH] OR "Bosnia and Herzegovina" [TIAB]) OR (Bulgaria [MESH] OR Bulgaria [TIAB]) OR (Croatia [MESH] OR Croatia [TIAB]) OR (Cyprus [MESH] OR Cyprus [TIAB]) OR (Czechia [MESH] OR Czechia [TIAB]) OR ("Czech Republic" [MESH] OR "Czech Republic" [TIAB]) OR (Denmark [MESH] OR Denmark [TIAB]) OR (Estonia [MESH] OR Estonia [TIAB]) OR (Finland [MESH] OR Finland [TIAB]) OR (France [MESH] OR France [TIAB]) OR (Germany [MESH] OR Germany [TIAB]) OR (Georgia [MESH] OR Georgia [TIAB]) OR (Greece [MESH] OR Greece [TIAB]) OR (Hungary [MESH] OR Hungary [TIAB]) OR (Iceland [MESH] OR Iceland [TIAB]) OR (Ireland [MESH] OR Ireland [TIAB]) OR (Israel [MESH] OR Israel [TIAB]) OR(Italy [MESH] OR Italy [TIAB]) OR (Kazakhstan [MESH] OR Kazakhstan [TIAB]) OR (Kosovo [MESH] OR Kosovo [TIAB]) OR (Latvia [MESH] OR Latvia [TIAB]) OR (Liechtenstein [MESH] OR Liechtenstein [TIAB]) OR (Lithuania [MESH] OR Lithuania

[TIAB]) OR (Luxembourg [MESH] OR Luxembourg [TIAB]) OR (Macedonia [MESH] OR Macedonia [TIAB]) OR (Malta [MESH] OR Malta [TIAB]) OR (Moldova [MESH] OR Moldova [TIAB]) OR (Monaco [MESH] OR Monaco [TIAB]) OR (Montenegro [MESH] OR Montenegro [TIAB]) OR (Netherlands [MESH] OR Netherlands [TIAB]) OR (Norway [MESH] OR Norway [TIAB]) OR (Poland [MESH] OR Poland [TIAB]) OR (Portugal [MESH] OR Portugal [TIAB]) OR (Romania [MESH] OR Romania [TIAB]) OR (Russia [MESH] OR Russia [TIAB]) OR ("Russian Federation" [MESH] OR "Russian Federation" [TIAB]) OR ("San Marino" [MESH] OR "San Marino" [TIAB]) OR (Serbia [MESH] OR Serbia [TIAB]) OR (Slovakia [MESH] OR Slovakia [TIAB]) OR (Slovenia [MESH] OR Slovenia [TIAB]) OR (Spain [MESH] OR Spain [TIAB]) OR (Sweden [MESH] OR Sweden [TIAB]) OR (Switzerland [MESH] OR Switzerland [TIAB])]) OR (Tajikistan [MESH] OR Tajikistan [TIAB]) OR (Turkey [MESH] OR Turkey [TIAB]) OR (Turkmenistan [MESH] OR Turkmenistan [TIAB]) OR (Ukraine [MESH] OR Ukraine [TIAB]) OR ("United Kingdom" [MESH] OR "United Kingdom" [TIAB]) OR (England [MESH] OR England [TIAB]) OR (Wales [MESH] OR Wales [TIAB]) OR (Scotland [MESH] OR Scotland [TIAB]) OR ("Northern Ireland" [MESH] OR "Northern Ireland" [TIAB]) OR (Faroes [MESH] OR Faroes [TIAB]) OR ("Vatican City" [MESH] OR "Vatican City" [TIAB])

Web of Science Query

("Streptococcus suis" and human) AND (Albania OR Andorra OR Armenia OR Austria OR Azerbaijan OR Belarus OR Belgium OR "Bosnia and Herzegovina" OR Bulgaria OR Croatia OR Cyprus OR Czechia OR "Czech Republic" OR Denmark OR Estonia OR Finland OR France

OR Germany OR Georgia OR Greece OR Hungary OR Iceland OR Ireland OR Israel OR Italy OR Kazakhstan OR Kosovo OR Latvia OR Liechtenstein OR Lithuania OR Luxembourg OR Macedonia OR Malta OR Moldova OR Monaco OR Montenegro OR Netherlands OR Norway OR Poland OR Portugal OR Romania OR Russia OR "Russian Federation" OR "San Marino" OR Serbia OR Slovakia OR Slovenia OR Spain OR Sweden OR Switzerland OR Tajikistan OR Turkey OR Turkmenistan OR Ukraine OR "United Kingdom" OR England OR Wales OR Scotland OR "Northern Ireland" OR Faroes OR "Vatican City")

Scopus Query

TITLE-ABS-KEY ("Streptococcus suis") AND TITLE-ABS-KEY (human) AND (TITLE-ABS-KEY (Albania OR Andorra OR Armenia OR Austria OR Azerbaijan OR Belarus OR Belgium OR "Bosnia and Herzegovina" OR Bulgaria OR Croatia OR Cyprus OR Czechia OR "Czech Republic" OR Denmark OR Estonia OR Finland OR France OR Germany OR Georgia OR Greece OR Hungary OR Iceland OR Ireland OR Israel OR Italy OR Kazakhstan OR Kosovo OR Latvia OR Liechtenstein OR Lithuania OR Luxembourg OR Macedonia OR Malta OR Moldova OR Monaco OR Montenegro OR Netherlands OR Norway OR Poland OR Portugal OR Romania OR Russia OR "Russian Federation" OR "San Marino" OR Serbia OR Slovakia OR Slovenia OR Spain OR Sweden OR Switzerland OR Tajikistan OR Turkey OR Turkmenistan OR Ukraine OR "United Kingdom" OR England OR Wales OR Scotland OR "Northern Ireland" OR Faroes OR "Vatican City")

III. Whole Genome Sequencing and Genome Assembly

Whole-genome sequencing

Out of 110 S. suis isolates identified in the reference laboratory survey, 24 had been previously sequenced, 40 isolates were not available for sequencing and 46 isolates were available and shared for sequencing (Appendix Figure 1). Isolates were initially grown overnight on sheep blood agar plates to test for contaminations and cultured overnight in Todd Hewitt Broth with 0.5% Yeast extract (THY), both at 37 ºC. Genomic DNA was extracted using the Wizard Genomic DNA Purification Kit (Promega, Madison, WI, USA) and DNA concentration was measured with Qubit using the dsDNA HS Assay Kit as well as the BR Assay Kit (Thermo Fisher Scientific Corporation, Eugene, OR, USA). Library preparation was performed using the KAPA HTP Library preparation kit (Roche, Basel, Switzerland). Pooled libraries were sequenced with the Reagent Kit v3 using paired-end Illumina MiSeq.

Genome Assembly

Unless otherwise specified, bioinformatic tools were used with the default settings. Raw sequencing reads were preprocessed using fastp v0.20.0 with the "--disable_length_filtering" flag (*1*) and Kraken2 with the MiniKraken2 database was used to confirm the taxonomical identification of the *S. suis* isolates (*2*). Draft genomes were assembled from the filtered Illumina reads using Shovill (v1.0.9 https://github.com/tseemann/shovill) with SPAdes v3.14.1 (*3*) and the quality of the draft genomes was evaluated with QUAST v5.0.2 (*4*). Genome annotation was carried out using Prokka v1.14.6 (*5*). *In silico* serotyping was performed feeding the processed Illumina reads into the *S. suis* serotyping pipeline (*6*). For isolate H143440710, the pipeline

could not distinguish between serotypes 1 and 14, therefore, the *cpsK* gene was Sanger sequenced using the BigDye™ Terminator v1.1 Cycle Sequencing Kit (Thermo Fisher Scientific Corporation, Eugene, OR, USA) to resolve its serotype. Primers used to distinguish between serotypes 1 and 14 are in Appendix Table 1.

IV. Incidence estimates of human *S. suis* infections in European countries with at least 5 cases between 2005 and 2013.

The incidence of human *S. suis* infections was estimated for all European countries with at least 5 *S. suis* cases reported in the survey or literature review cases between 2005 and 2013. The size of the population at risk was estimated based on the Eurostat Agricultural census (*81*). The agricultural census contained data regarding the number of agricultural holdings, pig specialised holdings and the size of the agricultural labour force for 2005, 2007, 2010 and 2013. The estimated population at risk for each year was calculated by multiplying the proportion of pig specialized agricultural holds by the total agricultural labour force (formula below). A 10% upper margin of error was then added to the average population at risk between 2005 and 2013 to account for butchers, hunters, slaughterhouse and meat factory workers not included in the agricultural census. The average size of the general population for each country between 2005 and 2013 was calculated by averaging the national census figures for 2005, 2007, 2010 and 2013 (*82–91*). When population size estimates were not available on national databases, the estimates from the OECD were used (*92*). The estimated incidence was calculated for both the total population and the population at risk (Appendix 4 Table). The data on *S. pneuomoniae* and *N.*

meningitidis incidence was reported by the European CDC (*93*). The average incidence between 2010 and 2013 (*S. pneumoniae*) and 2005 and 2013 (*N. meningitidis)* is displayed on (Appendix Table 6).

Formulas

$$Estimates\ on\ population\ at\ risk =$$

$$\left(\frac{number\ of\ pig\ holdings\ in\ a\ given\ country}{number\ of\ agricultural\ holdings\ in\ a\ given\ country}\right) x\ \ no.of\ persons\ in\ agricultural\ labour\ force$$

$$Estimated\ incidence\ of\ S.suis\ in\ the\ population$$

$$\left(\frac{number\ of\ confirmed\ cases\ of\ human\ S.suis\ during\ specified\ period}{size\ of\ population\ \ in\ specified\ period}\right) x\ 100{,}000\ persons$$

Appendix Table 1. Primers used in this study to distinguish between serotype 1 and 14.

Primer	DNA sequence
cps14K_forward	GCTATCAGATGCCCGTAATTATGGC
cps14K_reverse	GCCTAAAAACAAAACAGCAAAGGC

Appendix Table 2. Collected *S. suis* cases and metadata during the Survey study

	Isolate Metadata								Patient Metadata				
Isolate	Country	Year isolated	Year reported	Source	Test method	Serotype	ST	WGS	Sex	Age range, y	Clinical symptoms	Occupation	Ref
133899	Czech Republic	NA	NA	NA	API Strep, PCR	2	11	This study	NA	NA	NA	NA	NA
136829	Czech Republic	NA	NA	NA	API Strep, PCR	2	1	This study	NA	NA	NA	NA	NA
139809	Czech Republic	NA	NA	NA	API Strep, PCR	2	1	This study	M	NA	NA	Works with pigs	NA
143741	Czech Republic	2001	NA	CSF	API Strep, PCR	2	25	This study	M	66–70	Meningitis	Works with pigs	NA
147422	Czech Republic	2003	NA	CSF	API Strep, PCR	2	1	This study	M	56–60	Meningitis	Works with pigs	NA
148550	Czech Republic	2003	NA	CSF	API Strep, PCR	2	1	This study	M	61–65	Meningitis	Works with pigs	NA
148897	Czech Republic	2003	NA	CSF	API Strep, PCR	2	25	This study	F	61–65	Meningitis	NA	NA
150002	Czech Republic	2004	NA	Blood	API Strep, PCR	2	25	This study	M	31–35	NA	NA	NA
150808	Czech Republic	2005	NA	CSF	API Strep, PCR	2	1	This study	F	36–40	Meningitis	Works with pigs	NA
150968	Czech Republic	2011	NA	Blood	API Strep, PCR	2	1	This study	F	51–55	Meningitis	Work with pigs	NA
155567	Czech Republic	2012	NA	CSF	API Strep, PCR	2	1	This study	M	56–60	Meningitis/ septicemia	NA	NA
515/2012	Czech Republic	2011	NA	Blood	API Strep, PCR	7	29	This study	M	51–55	Septicemia	NA	NA
300/2014	Czech Republic	2012	NA	CSF	API Strep, PCR	2	1	This study	F	41–45	Septicemia	NA	NA
821/2014	Czech Republic	2014	NA	CSF	API Strep, PCR	2	1	This study	M	0–5	Meningitis/ septicemia	NA	NA
783/2015	Czech Republic	2015	NA	Blood	API Strep, PCR	2	NA*	This study	M	61–65	NA	NA	NA
84/2016	Czech Republic	2016	NA	NA	API Strep, PCR	2	1	This study	F	61–65	Septicemia	NA	NA
150687	Czech Republic	2005	NA	CSF	API Strep, PCR	2	1660	This study	F	51–55	Meningitis	NA	NA
16770	Germany	1998	NA	CSF	NA	2	1	This study	NA	NA	NA	NA	NA
18911	Germany	2001	NA	Blood	NA	2	1	This study	M	31–35	NA	NA	NA
19691	Germany	2003	NA	Blood	NA	2	1	This study	M	46–50	NA	NA	NA
20014	Germany	2004	NA	CSF	NA	2	1	This study	F	36–40	NA	NA	NA

Isolate	Isolate Metadata								Patient Metadata				Ref
	Country	Year isolated	Year reported	Source	Test method	Serotype	ST	WGS	Sex	Age range, y	Clinical symptoms	Occupation	
23674	Germany	2005	NA	Blood	NA	2	1	This study	M	26–30	NA	NA	NA
26556	Germany	2005	NA	CSF	NA	14	1	This study	M	56–60	NA	NA	NA
29429	Germany	2004	NA	NA	NA	14	1	This study	NA	NA	NA	Pig farmer	NA
35192	Germany	2008	NA	CSF	NA	2	19	This study	M	66–70	NA	NA	NA
36305	Germany	2008	NA	Blood	NA	2	19	This study	M	61–65	NA	NA	NA
45583	Germany	2010	NA	Blood	NA	2	1	This study	F	71–75	NA	NA	NA
46581	Germany	2010	NA	Blood	NA	2	1	This study	F	46–50	NA	NA	NA
15194	Germany	1990	NA	CSF	NA	2	1661	This study	NA	NA	NA	NA	NA
15459	Germany	NA	NA	NA	NA	2	1	This study	NA	NA	NA	NA	NA
15901	Germany	1994	NA	NA	NA	14	1	This study	NA	NA	NA	NA	NA
16064	Germany	1994	NA	NA	NA	2	1662	This study	NA	NA	NA	NA	NA
17387	Germany	1999	NA	CSF	NA	5	1663	This study	NA	NA	NA	NA	NA
18364	Germany	2000	NA	CSF	NA	2	1	This study	M	36–40	NA	NA	NA
18939	Germany	2001	NA	Blood	NA	2	1	This study	M	NA	NA	NA	NA
19514	Germany	2002	NA	NA	NA	2	1	This study	NA	NA	NA	NA	NA
21150	Germany	2004	NA	CSF	NA	14	1	This study	M	26–30	NA	NA	NA
82749	Germany	2018	NA	Blood	NA	5	1707	This study	M	41–45	NA	Pig farmer	NA
16318	Germany	1996	NA	NA	NA	NA	NA	NA	NA	NA	NA	NA	NA
19395	Germany	2002	NA	NA	NA	NA	NA	NA	NA	NA	NA	NA	NA
19396	Germany	2002	NA	NA	NA	NA	NA	NA	NA	NA	NA	NA	NA
48919	Germany	2011	NA	Blood	NA	NA	NA	NA	F	46–50	NA	NA	NA
4249	Denmark	NA	NA	Blood	Phenotypic methods	14	NA	NA	NA	NA	Sepsis	NA	NA
H48896	Denmark	2002	NA	Blood	Phenotypic methods	14	NA	NA	M	56–60	Sepsis	NA	NA
None	Denmark	NA	NA	CSF	Phenotypic methods	14	NA	NA	M	NA	Meningitis/ sepsis	NA	NA

	Isolate Metadata								Patient Metadata				
Isolate	Country	Year isolated	Year reported	Source	Test method	Serotype	ST	WGS	Sex	Age range, y	Clinical symptoms	Occupation	Ref
H55346	Denmark	2002	NA	Blood	Phenotypic methods	14	NA	NA	M	51–55	Sepsis	NA	NA
DK1	Denmark	2016	NA	Blood	NA	2	1	This study	F	51–55	NA	NA	NA
901131	Netherlands	1990	2012	CSF/ Blood	NA	2	134	*7*	M	51–55	Meningitis	NA	*7*
902409	Netherlands	1990	2012	NA	API Strep	2	1	*7*	M	36–40	Meningitis	NA	*7*
920694	Netherlands	1992	2012	CSF	API Strep	2	20	*7*	M	56–60	Meningitis	NA	*7*
931260	Netherlands	1993	2012	CSF	API Strep	2	20	*7*	M	NA	Meningitis	NA	*7*
940056	Netherlands	1994	2012	CSF/ Blood	API Strep	2	1	*7*	M	56–60	Meningitis	NA	*7*
940255	Netherlands	1994	2012	Blood	API Strep	2	20	*7*	M	51–55	Meningitis	Pig breeder	*7*
940430	Netherlands	1994	2012	CSF/ Blood	API Strep	2	1	*7*	M	51–55	Meningitis	NA	*7*
941372	Netherlands	1994	2012	CSF	API Strep	2	20	*7*	M	61–65	Meningitis	NA	*7*
950136	Netherlands	1995	2012	CSF	API Strep	2	20	*7*	M	51–55	Meningitis	NA	*7*
971344	Netherlands	1997	2012	CSF/ Blood	API Strep	2	1	*7*	M	41–45	Meningitis	Pig keeper	*7*
971724	Netherlands	1997	2012	Blood	API Strep	2	1	*7*	M	51–55	Meningitis	Butcher	*7*
2000343	Netherlands	2000	2008	CSF	API Strep	2	1	*7*	M	61–65	Meningitis	Pig farmer	*8*
2001171	Netherlands	2000	2012	Blood	API Strep	2	20	*7*	M	61–65	Meningitis	Pig keeper	*7*
2001946	Netherlands	2000	2008	CSF/ Blood	MALDI-TOF	2	1	*7*	M	56–60	Meningitis	Pig farmer	*8*
2011423	Netherlands	2001	2008	CSF	MALDI-TOF	2	1	*7*	M	46–50	Meningitis	Pig farmer	*8*
2012092	Netherlands	2001	2012	CSF	API Strep	2	20	*7*	M	31–35	Meningitis	Meat processing	*7*
2032008	Netherlands	2003	2012	CSF/ Blood	API Strep	2	20	*7*	M	56–60	Meningitis	Pig keeper	*7*
2051729	Netherlands	2005	2012	CSF/ Blood	API Strep	2	20	*7*	M	66–70	Meningitis	NA	*7*
2061238	Netherlands	2006	2012	CSF	API Strep	2	146	*7*	M	26–30	Meningitis	NA	*7*
2061410	Netherlands	2006	2012	CSF/ Blood	API Strep	2	1	*7*	M	36–40	Meningitis	NA	*7*
2071319	Netherlands	2007	2012	CSF	API Strep	2	1	*7*	M	46–50	Meningitis	Butcher	*7*
902035	Netherlands	1990	2012	CSF	API Strep	NA	NA	NA	F	0–5	Meningitis/ sepsis	NA	*7*
20120669	Netherlands	2007	2012	Blood	API Strep	NA	NA	NA	M	51–55	Meningitis	NA	*7*
2121473	Netherlands	2012	2012	CSF/ Blood	API Strep	NA	NA	NA	M	41–45	Meningitis/ sepsis	NA	*7*
2150651	Netherlands	2015	2012	Blood	API Strep	NA	NA	NA	M	56–60	Meningitis	NA	*7*
H17232068 7	United Kingdom	2017	NA	CSF	API RAPID ID32/ 16S rDNA seq	2	1708	This study	M	46–50	Meningitis	NA	NA

	Isolate Metadata								Patient Metadata				
Isolate	Country	Year isolated	Year reported	Source	Test method	Serotype	ST	WGS	Sex	Age range, y	Clinical symptoms	Occupation	Ref
H143440709	Ireland	2014	NA	CSF	API RAPID ID32/ 16S rDNA seq	14	1	This study	M	NA	Meningitis	NA	NA
H155260819	Ireland	2015	NA	Blood	API RAPID ID32/ 16S rDNA seq	2	1	This study	M	41–45	Septicemia	NA	NA
H143440711	Ireland	2014	NA	Blood	API RAPID ID32/ 16S rDNA seq	14	1	This study	M	NA	Meningitis	NA	NA
H104080146	United Kingdom	2010	NA	Blood	API RAPID ID32/ 16S rDNA seq	2	1	This study	M	76–80	Septicemia	NA	NA
H143440710	Ireland	2014	NA	Blood	API RAPID ID32/ 16S rDNA seq	14	1	This study	M	NA	Meningitis	NA	NA
SP1	Spain	2014	NA	NA	NA	2	1	This study	M	46–50	NA	Abattoir worker	NA
Spain2	Spain	NA	NA	NA	NA	NA	NA	NA	NA	NA	UTI	NA	NA
Spain3	Spain	NA	NA	Blood	NA	NA	NA	NA	NA	NA	Bacteremia	NA	NA
Spain4	Spain	NA	NA	Blood	NA	NA	NA	NA	NA	NA	Bacteremia	NA	NA
Spain5	Spain	2014	NA	NA	NA	NA	NA	NA	M	46–50	NA	NA	NA
Spain6	Spain	2018	NA	NA	NA	NA	NA	NA	M	61–65	NA	NA	NA
Spain7	Spain	NA	NA	Semen	NA	NA	NA	NA	M	NA	NA	NA	NA
Spain8	Spain	NA	NA	CSF	NA	NA	NA	NA	NA	NA	NA	NA	NA
2309	Poland	2000	2016	CSF	Vitek MS	2	1	NA	M	46–50	Meningitis	NA	*9*
3177	Poland	2003	2016	CSF	Vitek MS	2	1	NA	M	46–50	Meningitis	NA	*9*
3006	Poland	2004	2016	CSF/ Blood	Vitek MS	2	1	NA	M	46–50	Meningitis	Butcher	*9*
3899	Poland	2004	2016	CSF	Vitek MS	2	1	NA	M	46–50	Meningitis	Pig farmer	*9*
2610	Poland	2005	2016	CSF/ Blood	Vitek MS	2	1	NA	F	51–55	Meningitis	NA	*9*
3828	Poland	2005	2016	CSF	Vitek MS	2	1	NA	F	36–40	Meningitis	NA	*9*
626	Poland	2006	2016	Blood	Vitek MS	2	1	NA	M	36–40	Endocarditis	NA	*9*
584	Poland	2007	2016	CSF/ Blood	Vitek MS	2	1	NA	M	26–30	Meningitis	NA	*9*
3586	Poland	2008	2016	CSF/ Blood	Vitek MS	2	1	NA	M	66–70	Meningitis	NA	*9*
6571	Poland	2009	2016	CSF	Vitek MS	2	1	NA	M	36–40	Meningitis	NA	*9*
41	Poland	2010	2016	Blood	Vitek MS	2	1	NA	M	56–60	Meningitis	NA	*9*
1738	Poland	2010	2016	CSF	Vitek MS	2	1	NA	M	51–55	Meningitis	NA	*9*
4524	Poland	2010	2016	CSF	Vitek MS	2	1	NA	M	56–60	Meningitis	NA	*9*
6021	Poland	2010	2016	CSF	Vitek MS	2	1	NA	F	51–55	Meningitis	NA	*9*
5228	Poland	2011	2016	CSF/ Blood	Vitek MS	2	1	NA	M	31–35	Meningitis	NA	*9*
8719	Poland	2011	2016	CSF	Vitek MS	2	1	NA	M	56–60	Meningitis	NA	*9*

	Isolate Metadata								Patient Metadata				
Isolate	Country	Year isolated	Year reported	Source	Test method	Serotype	ST	WGS	Sex	Age range, y	Clinical symptoms	Occupation	Ref
174	Poland	2012	2016	CSF	Vitek MS	2	1	NA	M	61–65	Meningitis	NA	*9*
6708	Poland	2012	2016	CSF	Vitek MS	2	1	NA	M	46–50	Meningitis	NA	*9*
4011	Poland	2013	2016	CSF	Vitek MS	2	1	NA	M	56–60	Meningitis	NA	*9*
4364	Poland	2013	2016	CSF	Vitek MS	2	1	NA	F	51–55	Meningitis	NA	*9*
5604	Poland	2013	2016	CSF	Vitek MS	2	1	NA	M	61–65	Meningitis	NA	*9*

*To avoid case identification, the age of each patient is shown as a 5 year range. One of the 7 core genes used for MLST typing did not assemble; the other 6 genes had allele 1 suggesting this isolate belongs to CC1. CC, clonal complex; MLST, multilocus sequence typing; NA, not available; ST, sequence type; WGS, whole genome sequencing.

Appendix Table 3. Collected *S. suis* cases and metadata during the Systematic search study. To avoid case identification, the age of each patient is shown as a 5 year range*

	Isolate Metadata							Patient Metadata				
Isolate	Country	Year isolated	Year reported	Source	Serotype	ST	WGS	Sex	Age range, y	Clinical symptoms	Occupation	Ref
AUS01	Austria	NA	1999	CSF	NA	NA	NA	F	61–65	Meningitis; hearing loss	Pig farmer	*10*
CRO01	Croatia	2000	2002	Blood	NA	NA	NA	M	NA	Septic shock; Died	NA	*11*
CRO02	Croatia	2000	2002	CSF	NA	NA	NA	M	NA	Meningitis	NA	*11*
CZ01	Czech Republic	NA	2013	Blood	NA	NA	NA	M	61–65	Sepsis; spondylodiscitis	Pig farmer	*12*
MAC 724	Germany	NA	2002	Blood	2	1	NA	M	NA	Died	Butcher	*13*
BK52339	Germany	2012	2015	Blood	2	1	NA	M	NA	Septicemia; died	Hunter	*14*
TiHo441	Germany	2011	2015	Blood	2	1	NA	NA	NA	Septicemia	NA	*14*
TiHo224	Germany	NA	2005	Blood/heart	2	NA	NA	M	41–45	Endocarditis	NA	*15*
199	Germany	NA	2003	CSF/blood	NA	NA	NA	M	51–55	Sepsis; meningitis; hearing loss	Hunter	*16*
DE06	Germany	NA	2002	Blood	2	NA	NA	M	36–40	Septic shock	Truck driver (pork)	*17*
DE07	Germany	NA	2002	Pharyngeal swab	2	NA	NA	NA	NA	NA	Abattoir	*17*
DE08	Germany	NA	2002	Pharyngeal swab	2	NA	NA	NA	NA	NA	Abattoir	*17*
DE09	Germany	NA	2002	Pharyngeal swab	2	NA	NA	NA	NA	NA	Abattoir	*17*
DE10	Germany	NA	2002	Pharyngeal swab	2	NA	NA	NA	NA	NA	Meat processing factory	*17*
DE11	Germany	NA	2002	Pharyngeal swab	2	NA	NA	NA	NA	NA	Meat processing factory	*17*
DE12	Germany	NA	2002	Pharyngeal swab	2	NA	NA	NA	NA	NA	Meat processing factory	*17*

	Isolate Metadata							Patient Metadata				
Isolate	Country	Year isolated	Year reported	Source	Serotype	ST	WGS	Sex	Age range, y	Clinical symptoms	Occupation	Ref
DE13	Germany	NA	2002	Pharyngeal swab	2	NA	NA	NA	NA	NA	Meat processing factory	*17*
DE14	Germany	NA	2007	CSF/ blood	2	NA	NA	M	41–45	Meningitis; hearing loss	Butcher	*18*
DE15	Germany	NA	1997	CSF/ blood	NA	NA	NA	M	51–55	Meningitis, hearing loss	Hunter	*19*
DSM28762	Germany	2002	2021	Blood	2	19	Yes	NA	NA	NA	NA	*20*
11611	Germany	NA	2013	NA	2	1	Yes	NA	NA	NA	NA	*21*
DK01	Denmark	2006	2009	CSF/ blood	14	NA	NA	M	51–55	Meningitis; spondylodiscitis, bacteremia	Pig farmer	*22*
FR01	France	NA	1996	CSF/ blood	NA	NA	NA	M	26–30	Meningitis	Butcher	*23*
FR02	France	1991	1992	CSF	NA	NA	NA	M	56–60	Meningitis	Meat processing factory	*24*
FR03	France	NA	2001	CSF	2	NA	NA	M	56–60	Meningitis, hearing loss	Pig breeder and hunter	*25*
FR04	France	1994	1996	Blood	2	NA	NA	M	36–40	Endocarditis	Butcher	*26*
FR05	France	1996	1998	CSF/ blood	2	1	NA	M	46–50	Meningitis; septic shock;	Butcher	*27*
FR06	France	NA	2008	CSF	NA	NA	NA	M	31–35	Meningitis; visual impairment	Lorry driver	*28*
FR07	France	NA	2003	Blood	NA	NA	NA	NA	NA	Bacteremia; septic shock; died	Hunter	*29*
GRE01	Greece	NA	2015	CSF/ blood	NA	NA	NA	M	31–35	Fever; partial hearing loss	Pig farmer	*30*
GRE02	Greece	NA	2005	CSF/ blood	2	NA	NA	F	61–65	Meningitis; sepsis; hearing loss	NA	*31*
GRE03	Greece	NA	2006	Blood	NA	NA	NA	M	56–60	Endocarditis	Butcher and farmer	*32*
HUN01	Hungary	NA	2020	Blood	2	NA	NA	M	31–35	Died; sepsis	Butcher	*33*
HUN02	Hungary	NA	2020	Blood	NA	NA	NA	M	NA	NA	NA	*34*
HUN03	Hungary	NA	2020	Blood	NA	NA	NA	M	NA	NA	NA	*34*
HUN04	Hungary	NA	2020	Blood	NA	NA	NA	M	NA	NA	NA	*34*
HUN05	Hungary	NA	2020	Blood	NA	NA	NA	M	NA	NA	NA	*34*
HUN06	Hungary	NA	2020	Blood	NA	NA	NA	M	NA	NA	NA	*34*
HUN07	Hungary	NA	2020	Blood	NA	NA	NA	M	NA	NA	NA	*34*
HUN08	Hungary	NA	2020	Blood	NA	NA	NA	M	NA	NA	NA	*34*
HUN09	Hungary	NA	2020	Blood	NA	NA	NA	M	NA	NA	NA	*34*
HUN10	Hungary	NA	2020	Blood	NA	NA	NA	M	NA	NA	NA	*34*
HUN11	Hungary	NA	2020	Blood	NA	NA	NA	M	NA	NA	NA	*34*
HUN12	Hungary	NA	2020	Blood	NA	NA	NA	M	NA	NA	NA	*34*
HUN13	Hungary	NA	2020	Blood	NA	NA	NA	M	NA	NA	NA	*34*
HUN14	Hungary	NA	2020	Blood	NA	NA	NA	M	NA	NA	NA	*34*

	Isolate Metadata							Patient Metadata				
Isolate	Country	Year isolated	Year reported	Source	Serotype	ST	WGS	Sex	Age range, y	Clinical symptoms	Occupation	Ref
HUN15	Hungary	NA	2020	Blood	NA	NA	NA	M	NA	NA	NA	*34*
HUN16	Hungary	NA	2020	Blood	NA	NA	NA	M	NA	NA	NA	*34*
HUN17	Hungary	NA	2020	Blood	NA	NA	NA	M	NA	NA	NA	*34*
HUN18	Hungary	NA	2020	Blood	NA	NA	NA	M	NA	NA	NA	*34*
HUN19	Hungary	NA	2020	Blood	NA	NA	NA	M	NA	NA	NA	*34*
HUN20	Hungary	NA	2020	Blood	NA	NA	NA	M	NA	NA	NA	*34*
HUN21	Hungary	NA	2020	Blood	NA	NA	NA	M	NA	NA	NA	*34*
HUN22	Hungary	NA	2020	CSF/ blood	NA	NA	NA	F	NA	NA	NA	*34*
HUN23	Hungary	NA	2020	CSF/ blood	NA	NA	NA	F	NA	NA	NA	*34*
HUN24	Hungary	NA	2020	CSF/ blood	NA	NA	NA	F	NA	NA	NA	*34*
HUN25	Hungary	NA	2020	CSF/ blood	2	NA	NA	F	NA	NA	NA	*34*
HUN26	Hungary	NA	2020	CSF/ blood	2	NA	NA	F	NA	NA	NA	*34*
HUN27	Hungary	NA	2020	CSF/ blood	2	NA	NA	F	NA	NA	NA	*34*
HUN28	Hungary	NA	2020	CSF/ blood	2	NA	NA	F	NA	NA	NA	*34*
HUN29	Hungary	NA	2020	CSF/ blood	2	NA	NA	F	NA	NA	NA	*34*
HUN30	Hungary	NA	2020	CSF/ blood	2	NA	NA	F	NA	NA	NA	*34*
HUN31	Hungary	NA	2020	CSF/ blood	2	NA	NA	F	NA	NA	NA	*34*
HUN32	Hungary	NA	2020	CSF/ blood	2	NA	NA	F	NA	NA	NA	*34*
HUN33	Hungary	NA	2020	CSF/ blood	2	NA	NA	F	NA	NA	NA	*34*
HUN34	Hungary	NA	2020	CSF/ blood	2	NA	NA	F	NA	NA	NA	*34*
HUN35	Hungary	NA	2020	CSF/ blood	14	NA	NA	F	NA	NA	NA	*34*
HUN36	Hungary	NA	2019	CSF/ blood	NA	NA	NA	M	46–50	Meningitis	Hunter	*35*
IRE01	Ireland	NA	1995	NA	NA	NA	NA	M	21–25	Sepsis, died	Pig farmer	*36*
ITA01	Italy	NA	2007	CSF	NA	1	NA	M	26–30	Meningitis	Butcher	*37*
SsRC-1	Italy	2016	2016	Blood	2	1	Yes	M	51–55	Septic shock, died	Pig farmer	*38*
ITA03	Italy	2007	2008	CSF	2	1	NA	M	66–70	Meningitis	NA	*39*
ITA04	Italy	NA	1995	CSF	NA	NA	NA	NA	51–55	Meningitis	Farmer	*40*
ITA05	Italy	NA	2010	CSF	NA	NA	NA	F	46–50	Meningitis	Caregiver	*41*
861160	Netherlands	1986	2012	CSF	2	20	Yes	M	36–40	Meningitis	NA	*42*
870040	Netherlands	1987	2012	CSF	2	1	Yes	M	26–30	Meningitis	NA	*42*
890267	Netherlands	1989	2012	CSF	2	1	Yes	M	46–50	Meningitis	NA	*42*
NL01	Netherlands	2006	2012	CSF/ blood	NA	NA	NA	M	56–60	Meningitis, hearing loss	Meat processing factory	*43*
NL26	Netherlands	2001	2008	CSF	NA	NA	NA	F	31–35	Meningitis	Butcher	*44*
NL27	Netherlands	2007	2015	CSF/ blood	NA	NA	NA	M	61–65	Meningitis, hearing loss	Pig farmer	*45*
NL28	Netherlands	2008	2015	CSF/ blood	NA	NA	NA	M	36–40	Meningitis, hearing loss	Pig farmer	*45*
NL29	Netherlands	2011	2015	CSF/ blood	NA	NA	NA	M	56–60	Meningitis, hearing loss	Abattoir	*45*
NL30	Netherlands	2012	2015	CSF/ blood	NA	NA	NA	M	76–80	Meningitis, hearing loss	Pig farmer	*45*
NL31	Netherlands	2015	2015	Blood	2	NA	NA	M	56–60	Meningitis	Butcher	*45*

	Isolate Metadata							Patient Metadata				
Isolate	Country	Year isolated	Year reported	Source	Serotype	ST	WGS	Sex	Age range, y	Clinical symptoms	Occupation	Ref
NL32	Netherlands	1989	1990	Blood	NA	NA	NA	M	26–30	Sepsis	Pig weigher	*46*
POL22	Poland	NA	2013	CSF/ blood	NA	NA	NA	M	31–35	Meningitis	Butcher	*47*
POR01	Portugal	2011	2014	CSF/ blood	NA	NA	NA	M	41–45	Meningitis	Pork griller	*48*
POR02	Portugal	NA	2008	CSF	NA	NA	NA	M	31–35	Meningitis	NA	*49*
POR03	Portugal	NA	2005	NA	NA	NA	NA	M	46–50	Meningitis	Butcher	*50*
POR04	Portugal	NA	2017	CSF/ blood	NA	NA	NA	M	46–50	Meningitis, hearing loss	Butcher	*51*
SER01	Serbia	2003	2005	CSF/ blood	NA	NA	NA	M	NA	Meningitis, hearing loss	Pig farmer	*52*
SER02	Serbia	2003	2005	CSF	NA	NA	NA	M	NA	Meningitis, hearing loss	Pig farmer	*52*
SER03	Serbia	2003	2005	CSF	NA	NA	NA	M	NA	Meningitis, hearing loss	Pig farmer	*52*
SER04	Serbia	2003	2005	CSF	NA	NA	NA	M	NA	Meningitis, hearing loss	NA	*52*
SER05	Serbia	2003	2005	CSF	NA	NA	NA	M	NA	Meningitis	NA	*52*
SP01	Spain	NA	2006	Blood	NA	NA	NA	M	56–60	Endocarditis, spondylodiscitis	Restaurant owner	*53*
SP02	Spain	NA	2009	Blood	2	NA	NA	M	66–70	Endocarditis	Helped at pig farm	*54*
SP03	Spain	NA	2012	Blood	NA	NA	NA	M	61–65	Meningitis	NA	*55*
SP04	Spain	1999	2001	CSF/ blood	NA	NA	NA	M	26–30	Meningitis	Butcher	*56*
SP05	Spain	1999	2001	CSF	NA	NA	NA	M	21–25	Meningitis, hearing loss	Butcher	*56*
SP06	Spain	2012	2014	CSF/ blood	2	3	NA	M	56–60	Meningitis, septic shock	NA	*57*
SP07	Spain	NA	2007	CSF/ blood	NA	NA	NA	M	31–35	Meningitis, hearing loss	Chicken farmer	*58*
SP08	Spain	NA	2008	CSF/ blood	NA	NA	NA	F	26–30	Meningitis	Butcher	*59*
SP09	Spain	NA	2016	Blood	2	NA	NA	M	51–55	Septic shock	Pig farmer	*60*
SP10	Spain	2004	2006	CSF/ blood	NA	NA	NA	M	36–40	Meningitis	Pig farmer	*61*
SP11	Spain	NA	2012	Blood	NA	NA	NA	M	31–35	Meningitis, hearing loss	Pig farmer	*62*
SP12	Spain	NA	2009	CSF	NA	NA	NA	F	81–85	Meningitis, spondylodiscitis	NA	*63*
SP13	Spain	NA	1994	NA	NA	NA	NA	F	56–60	Sacrolitis	NA	*64*
SP14	Spain	NA	2011	CSF/ blood	2	NA	NA	F	31–35	Meningitis, hearing loss	Pig farmer	*65*
SP15	Spain	NA	2005	CSF/ blood	2	NA	NA	M	26–30	Meningitis	Meat processing factory	*66*
SP16	Spain	NA	2012	Blood	2	3	NA	M	36–40	Meningitis, visual impairment	Pig farmer	*67*
SP17	Spain	1998	1998	CSF/ blood	NA	NA	NA	M	46–50	Meningitis, hearing loss	Pig farmer	*68*

	Isolate Metadata							Patient Metadata				
Isolate	Country	Year isolated	Year reported	Source	Serotype	ST	WGS	Sex	Age range, y	Clinical symptoms	Occupation	Ref
SP18	Spain	1998	1998	CSF	NA	NA	NA	M	46–50	Meningitis	Butcher	*68*
SP19	Spain	1997	1997	CSF/ blood	NA	NA	NA	M	51–55	Meningitis	Pig farmer	*69*
SP20	Spain	2014	2018	NA	2	1	NA	M	46–50	Meningitis, arthritis	Hunter	*70*
SP21	Spain	2013	2021	CSF/ blood	NA	NA	NA	M	46–50	Meningitis	Butcher	*71*
SP22	Spain	2014	2021	CSF	NA	NA	NA	M	46–50	Meningitis	Pig farmer	*71*
SP23	Spain	2018	2021	Blood	NA	NA	NA	M	61–65	Endocarditis	Pig farmer	*71*
SP24	Spain	2018	2021	Semen	NA	NA	NA	M	46–50	Orchitis	NA	*71*
SP25	Spain	2019	2021	CSF	NA	NA	NA	M	41–45	Meningitis	Pig farmer	*71*
SP26	Spain	2019	2021	Blood/ synovial fluid	NA	NA	NA	M	61–65	Sepsis/ arthritis	Pig farmer	*71*
SP27	Spain	2019	2021	Synovial fluid	NA	NA	NA	M	36–40	Prosthetic hip infection	Pig farmer	*71*
SP28	Spain	NA	2014	CSF	NA	NA	NA	M	61–65	Meningitis	NA	*72*
SWE01	Sweden	NA	2014	Blood/ synovial fluid	5	NA	NA	M	61–65	Arthritis	Pig farmer	*73*
GE-034	Switzerland	NA	2020	Blood	14	1	Yes	F	41–45	Septic shock	NA	*74*
UK01	United Kingdom	NA	1991	CSF/ blood	NA	NA	NA	M	56–60	Meningitis, died	Meat porter	*75*
UK02	United Kingdom	NA	1992	CSF/ blood	NA	NA	NA	M	46–50	Meningitis, visual impairment	Pig farmer	*76*
UK03	United Kingdom	1999	2001	Blood	14	NA	NA	M	26–30	Septicemia, died	Pig farmer	*77*
UK04	United Kingdom	NA	1990	Blood	NA	NA	NA	F	41–45	Sepsis	Pig farmer	*78*

*NA, not available; ST, sequence type; WGS, whole genome sequencing.

Appendix Table 4. Number of reported human *S. suis* infections in the Public Health England zoonosis reports (*79*)

Years	No. of isolates
1991–2000	20
2001	1
2002	1
2003	1
2004	0
2005	2
2006	2
2007	2
2008	7
2009	2
2010	4
2011	1
2012	3
2013	3
2014	3
2015	4
2016	1
2017	4
Total	61

Appendix Table 5. Number of reported human *S. suis* infections in the National Institute for Public Health and Environment of the Netherlands. To avoid case identification, the age of each patient is shown as a 5 year range NA stands for missing information

	Isolate Metadata		Patient Metadata			
Isolate	Year isolated	Source	Sex	Age range, y	Clinical symptoms	Ref
NL33	1990	CSF	M	41–45	NA	80
NL34	1990	Blood	M	66–70	NA	80
NL35	1992	Blood	M	71–75	NA	80
NL36	1993	Blood	NA	51–55	NA	80
NL37	1996	Throat swab	M	36–40	NA	80
NL38	1996	NA	M	71–75	NA	80
NL39	1996	Blood	M	51–55	Meningitis	80
NL40	1999	Blood	M	66–70	NA	80
NL41	2004	Blood	M	0–5	NA	80
NL42	2006	CSF/ blood	M	61–65	NA	80
NL43	2006	Blood	M	56–60	NA	80
NL44	2007	Urine	F	0–5	Urinary tract infection	80
NL45	2008	Dialysate	F	56–60	Peritonitis	80
NL46	2008	Urine	F	41–45	NA	80
NL47	2008	Bilious abdominal fluid	M	86–90	NA	80
NL48	2009	Wound culture	F	51–55	NA	80
NL49	2009	Wound culture	F	36–40	NA	80
NL50	2009	Wound culture	M	36–40	NA	80
NL51	2010	Urine	F	21–25	Urinary tract infection	80
NL52	2010	Abscess fluid	M	26–30	NA	80
NL53	2011	Urine	F	66–70	NA	80
NL54	2011	Pus	M	71–75	NA	80
NL55	2011	CSF/ blood	M	46–50	NA	80
NL56	2011	Urethra swab	M	66–70	NA	80
NL57	NA	Wound culture	F	NA	Sepsis	80

Appendix Table 6. Incidence estimates of human *S. suis* infections in European countries with at least 5 cases during 2005–2013*

Country	Population, thousands†	Estimated at-risk population, thousands	No. of *S. suis* infections	Incidence in total population /100,000 persons‡	Incidence in at-risk population, /100,000 persons‡	*S. pneumoniae* incidence in total population /100,000 persons	*N. meningitidis* incidence in total population /100,000 persons
Czech Republic	10,393.68	46.05	7	0.0075	1.689	3.19	0.69
Germany	81,793.72	189.53	12	0.0016	0.703	NA	0.55
Hungary	10,021.71	509.01	36	0.0399	0.786	1.52	0.42
Netherlands	16,507.50	24.72	11	0.0074	4.945	14.86	0.93
Poland*	38,324.00	1,242.46	18	0.0052	0.161	1.10	0.67
Spain	45,323.88	189.92	12	0.0029	0.702	5.84	1.09

*NA, not available.
†Used OECD estimates for total population
‡Rough estimates of incidence and demographics of human *Streptococcus suis* infection reported to national reference laboratories during 2005–2013.

Appendix Table 7. Sequences used in this study and their accession numbers.

Strain	Bioproject	Genbank	Biosample	SRA Illumina	Reference
This study					
133899	PRJNA917482	JARATX000000000	SAMN32539671	SRR26047323	This study
136829	PRJNA917482	JAVMBI000000000	SAMN32539672	SRR26047322	This study
139809	PRJNA917482	JARATW000000000	SAMN32539673	SRR26047311	This study
143741	PRJNA917482	JARATV000000000	SAMN32539674	SRR26047300	This study
147422	PRJNA917482	JARATU000000000	SAMN32539675	SRR26047289	This study
148550	PRJNA917482	JARATT000000000	SAMN32539676	SRR26047282	This study
148897	PRJNA917482	JARATS000000000	SAMN32539677	SRR26047281	This study
150002	PRJNA917482	JARATR000000000	SAMN32539678	SRR26047280	This study
150808	PRJNA917482	JARATQ000000000	SAMN32539679	SRR26047279	This study
150968	PRJNA917482	JARATP000000000	SAMN32539680	SRR26047278	This study
155567	PRJNA917482	JARATO000000000	SAMN32539681	SRR26047321	This study
515/2012	PRJNA917482	JARATN000000000	SAMN32539682	SRR26047320	This study
300/2014	PRJNA917482	JARATM000000000	SAMN32539683	SRR26047319	This study
821/2014	PRJNA917482	JARATL000000000	SAMN32539684	SRR26047318	This study
783/2015	PRJNA917482	JAVMBJ000000000	SAMN32539685	SRR26047317	This study
84/2016	PRJNA917482	JARATK000000000	SAMN32539686	SRR26047316	This study
150687	PRJNA917482	JAVMBK000000000	SAMN32539687	SRR26047315	This study
16770	PRJNA917482	JARATJ000000000	SAMN32539688	SRR26047314	This study
18911	PRJNA917482	JARATI000000000	SAMN32539689	SRR26047313	This study
19691	PRJNA917482	JAVMBL000000000	SAMN32539690	SRR26047312	This study
20014	PRJNA917482	JARATH000000000	SAMN32539691	SRR26047310	This study
23674	PRJNA917482	JARATG000000000	SAMN32539692	SRR26047309	This study
26556	PRJNA917482	JARATF000000000	SAMN32539693	SRR26047308	This study
29429	PRJNA917482	JAVMBM000000000	SAMN32539694	SRR26047307	This study
35192	PRJNA917482	JARATE000000000	SAMN32539695	SRR26047306	This study
36305	PRJNA917482	JARATD000000000	SAMN32539696	SRR26047305	This study
45583	PRJNA917482	JARATC000000000	SAMN32539697	SRR26047304	This study
46581	PRJNA917482	JARATB000000000	SAMN32539698	SRR26047303	This study
15194	PRJNA917482	JARATA000000000	SAMN32539699	SRR26047302	This study
15459	PRJNA917482	JARASZ000000000	SAMN32539700	SRR26047301	This study
15901	PRJNA917482	JARASY000000000	SAMN32539701	SRR26047299	This study
16064	PRJNA917482	JARASX000000000	SAMN32539702	SRR26047298	This study
17387	PRJNA917482	JARASW000000000	SAMN32539703	SRR26047297	This study
18364	PRJNA917482	JAVMBN000000000	SAMN32539704	SRR26047296	This study
18939	PRJNA917482	JARASV000000000	SAMN32539705	SRR26047295	This study
19514	PRJNA917482	JARASU000000000	SAMN32539706	SRR26047294	This study
21150	PRJNA917482	JARAST000000000	SAMN32539707	SRR26047293	This study
82749	PRJNA917482	JARASS000000000	SAMN32539708	SRR26047292	This study
DK1	PRJNA917482	JARASR000000000	SAMN32539709	SRR26047291	This study
H172320687	PRJNA917482	JARASQ000000000	SAMN32539710	SRR26047290	This study
H143440709	PRJNA917482	JARASP000000000	SAMN32539711	SRR26047288	This study
H155260819	PRJNA917482	JARASO000000000	SAMN32539712	SRR26047287	This study
H143440711	PRJNA917482	JARASN000000000	SAMN32539713	SRR26047286	This study
H104080146	PRJNA917482	JARASM000000000	SAMN32539714	SRR26047285	This study
H143440710	PRJNA917482	JARASL000000000	SAMN32539715	SRR26047284	This study

Strain	Bioproject	Genbank	Biosample	SRA Illumina	Reference
SP1	PRJNA917482	JARASK000000000	SAMN32539716	SRR26047283	This study
Public Genomes					
861160	PRJEB11219	CZEH00000000	SAMEA3595177	ERR1055554	*94*
870040	PRJEB11219	CZDK00000000	SAMEA3595178	ERR1055555	*94*
890267	PRJEB11219	CZDM00000000	SAMEA3595179	ERR1055556	*94*
901131	PRJEB11219	CZEW00000000	SAMEA3595180	ERR1055557	*94*
902409	PRJEB11219	CZDT00000000	SAMEA3595181	ERR1055558	*94*
920694	PRJEB11219	CZES00000000	SAMEA3595182	ERR1055559	*94*
931260	PRJEB11219	CZDW00000000	SAMEA3595183	ERR1055560	*94*
940056	PRJEB11219	CZEU00000000	SAMEA3595184	ERR1055561	*94*
940255	PRJEB11219	CZDN00000000	SAMEA3595185	ERR1055562	*94*
940430	PRJEB11219	CZDX00000000	SAMEA3595186	ERR1055563	*94*
941372	PRJEB11219	CZEJ00000000	SAMEA3595187	ERR1055564	*94*
950136	PRJEB11219	CZDZ00000000	SAMEA3595188	ERR1055565	*94*
971344	PRJEB11219	CZDL00000000	SAMEA3595189	ERR1055566	*94*
971724	PRJEB11219	CZEG00000000	SAMEA3595190	ERR1055567	*94*
2000343	PRJEB11219	CZET00000000	SAMEA3595191	ERR1055568	*94*
2001171	PRJEB11219	CZDY00000000	SAMEA3595192	ERR1055569	*94*
2001946	PRJEB11219	CZDO00000000	SAMEA3595193	ERR1055570	*94*
2011423	PRJEB11219	CZEO00000000	SAMEA3595194	ERR1055571	*94*
2012092	PRJEB11219	CZEK00000000	SAMEA3595195	ERR1055572	*94*
2032008	PRJEB11219	CZEV00000000	SAMEA3595196	ERR1055573	*94*
2051729	PRJEB11219	CZEE00000000	SAMEA3595197	ERR1055574	*94*
2061238	PRJEB11219	CZDQ00000000	SAMEA3595198	ERR1055575	*94*
2061410	PRJEB11219	CZDV00000000	SAMEA3595199	ERR1055576	*94*
2071319	PRJEB11219	CZEQ00000000	SAMEA3595200	ERR1055577	*94*
SsRC-1	PRJNA445803	PYUF00000000	SAMN08799542	NA	*95*
GE-034	pubMLST	pubMLST	pubMLST	pubMLST	*96*
DSM28762	PRJEB45445	NA	SAMEA8942198	ERR6131134	*97*
11611	PRJNA171412	ALKR00000000	SAMN02470636	NA	*98*

References

1. Chen S, Zhou Y, Chen Y, Gu J. fastp: an ultra-fast all-in-one FASTQ preprocessor. Bioinformatics. 2018;34:i884–90. PubMed https://doi.org/10.1093/bioinformatics/bty560

2. Wood DE, Lu J, Langmead B. Improved metagenomic analysis with Kraken 2. Genome Biol. 2019;20:257. PubMed https://doi.org/10.1186/s13059-019-1891-0

3. Prjibelski A, Antipov D, Meleshko D, Lapidus A, Korobeynikov A. Using SPAdes de novo assembler. Curr Protoc Bioinformatics. 2020;70:e102. PubMed https://doi.org/10.1002/cpbi.102

4. Gurevich A, Saveliev V, Vyahhi N, Tesler G. QUAST: quality assessment tool for genome assemblies. Bioinformatics. 2013;29:1072–5. PubMed https://doi.org/10.1093/bioinformatics/btt086

5. Seemann T. Prokka: rapid prokaryotic genome annotation. Bioinformatics. 2014;30:2068–9. PubMed https://doi.org/10.1093/bioinformatics/btu153

6. Athey TBT, Teatero S, Lacouture S, Takamatsu D, Gottschalk M, Fittipaldi N. Determining *Streptococcus suis* serotype from short-read whole-genome sequencing data. BMC Microbiol. 2016;16:162. PubMed https://doi.org/10.1186/s12866-016-0782-8

7. Willemse N, Howell KJ, Weinert LA, Heuvelink A, Pannekoek Y, Wagenaar JA, et al. An emerging zoonotic clone in the Netherlands provides clues to virulence and zoonotic potential of Streptococcus suis. Sci Rep. 2016;6:28984. PubMed https://doi.org/10.1038/srep28984

8. van de Beek D, Spanjaard L, de Gans J. *Streptococcus suis* meningitis in the Netherlands. J Infect. 2008;57:158–61. PubMed https://doi.org/10.1016/j.jinf.2008.04.009

9. Bojarska A, Molska E, Janas K, Skoczyńska A, Stefaniuk E, Hryniewicz W, et al. *Streptococcus suis* in invasive human infections in Poland: clonality and determinants of virulence and antimicrobial resistance. Eur J Clin Microbiol Infect Dis. 2016;35:917–25. PubMed https://doi.org/10.1007/s10096-016-2616-x

10. Spiss HK, Kofler M, Hausdorfer H, Pfausler B, Schmutzhard E. *Streptococcus suis* meningitis and neurophysiology of the acoustic system. First case report from Austria [in German]. Nervenarzt. 1999;70:738–41. PubMed https://doi.org/10.1007/s001150050503

11. Kopić J, Paradžik MT, Pandak N. *Streptococcus suis* infection as a cause of severe illness: 2 cases from Croatia. Scand J Infect Dis. 2002;34:683–4. PubMed https://doi.org/10.1080/00365540210147769

12. Smetana J, Machac J, Prasil P, Bostikova V, Plisek S, Kosina P, et al. Spondylodiscitis with Abscess Formation in Paravertebral Muscles due to *Streptococcus suis*—a Case Report. Ces Slov Neurol Neurochir. 2013;76:769–72.

13. Baums CG, Verkühlen GJ, Rehm T, Silva LMG, Beyerbach M, Pohlmeyer K, et al. Prevalence of *Streptococcus suis* genotypes in wild boars of Northwestern Germany. Appl Environ Microbiol. 2007;73:711–7. PubMed https://doi.org/10.1128/AEM.01800-06

14. Eisenberg T, Hudemann C, Hossain HM, Hewer A, Tello K, Bandorski D, et al. Characterization of Five Zoonotic *Streptococcus suis* Strains from Germany, Including One Isolate from a Recent Fatal Case of Streptococcal Toxic Shock-Like Syndrome in a Hunter. J Clin Microbiol. 2015;53:3912–5. PubMed https://doi.org/10.1128/JCM.02578-15

15. Heidt MC, Mohamed W, Hain T, Vogt PR, Chakraborty T, Domann E. Human infective endocarditis caused by *Streptococcus suis* serotype 2. J Clin Microbiol. 2005;43:4898–901. PubMed https://doi.org/10.1128/JCM.43.9.4898-4901.2005

16. Rosenkranz M, Elsner HA, Stürenburg HJ, Weiller C, Röther J, Sobottka I. *Streptococcus suis* meningitis and septicemia contracted from a wild boar in Germany. J Neurol. 2003;250:869–70. PubMed https://doi.org/10.1007/s00415-003-1103-3

17. Strangmann E, Fröleke H, Kohse KP. Septic shock caused by *Streptococcus suis*: case report and investigation of a risk group. Int J Hyg Environ Health. 2002;205:385–92. PubMed https://doi.org/10.1078/1438-4639-00165

18. Braun S, Jechart G, Emmerling U, Ehret W. Bakterielle Meningitis durch *Streptococcus suis.* Dtsch Med Wochenschr. 2007;132:1098–100. PubMed https://doi.org/10.1055/s-2007-979385

19. Grebe T, Bergenthal D, Fahr AM, Scheja HW. Erwachsenen-Meningitis durch *Streptococcus suis* Typ 2. Dtsch Med Wochenschr. 1997;122:1244–7. PubMed https://doi.org/10.1055/s-2008-1047754

20. Werinder A, Aspán A, Söderlund R, Backhans A, Sjölund M, Guss B, et al. Whole-Genome Sequencing Evaluation of MALDI-TOF MS as a Species Identification Tool for *Streptococcus suis.* J Clin Microbiol. 2021;59:e0129721. PubMed https://doi.org/10.1128/JCM.01297-21

21. Chen C, Zhang W, Zheng H, Lan R, Wang H, Du P, et al. Minimum core genome sequence typing of bacterial pathogens: a unified approach for clinical and public health microbiology. J Clin Microbiol. 2013;51:2582–91. PubMed https://doi.org/10.1128/JCM.00535-13

22. Poggenborg R, Gaïni S, Kjaeldgaard P, Christensen JJ. *Streptococcus suis*: meningitis, spondylodiscitis and bacteraemia with a serotype 14 strain. Scand J Infect Dis. 2008;40:346–9. PubMed https://doi.org/10.1080/00365540701716825

23. Bezian MC, Diallo BS, Chachia A, Gabinski C, Beylot J. Fulminant purpura during *Streptococcus suis* meningitis and septicaemia. Med Mal Infect. 1996;26:349–51. https://doi.org/10.1016/S0399-077X(96)80212-5

24. Dupas D, Vignon M, Géraut C. *Streptococcus suis* meningitis. A severe noncompensated occupational disease. J Occup Med. 1992;34:1102–5. PubMed https://doi.org/10.1097/00043764-199211000-00013

25. Durand F, Périno CL, Recule C, Brion JP, Kobish M, Guerber F, et al. Bacteriological diagnosis of *Streptococcus suis* meningitis. Eur J Clin Microbiol Infect Dis. 2001;20:519–21. PubMed https://doi.org/10.1007/PL00011299

26. Tayoro J, Besnier JM, Laudat P, Cattier B, Choutet P. Infective endocarditis due to *Streptococcus suis* serotype 2. Eur J Clin Microbiol Infect Dis. 1996;15:765–6. PubMed https://doi.org/10.1007/BF01691970

27. François B, Gissot V, Ploy MC, Vignon P. Recurrent septic shock due to *Streptococcus suis.* J Clin Microbiol. 1998;36:2395–2395. PubMed https://doi.org/10.1128/JCM.36.8.2395-2395.1998

28. Bahloul H, Mofredj A, Mrabet A, Gineyt G, Rousselier P. Méningite à *Streptococcus suis* secondaire à une contamination orale? Med Mal Infect. 2008;38:281–2. PubMed https://doi.org/10.1016/j.medmal.2008.01.002

29. Pedroli S, Kobisch M, Beauchet O, Chaussinand JP, Lucht F. *Streptococcus suis* bacteremia [in French]. Presse Med. 2003;32:599–601. PubMed

30. Chatzopoulou M, Voulgaridou I, Papalas D, Vasiliou P, Tsiakalou M. Third Case of *Streptococcus suis* Infection in Greece. Case Rep Infect Dis. 2015;2015:505834. PubMed https://doi.org/10.1155/2015/505834

31. Mazokopakis EE, Kofteridis DP, Papadakis JA, Gikas AH, Samonis GJ. First case report of *Streptococcus suis* septicaemia and meningitis from Greece. Eur J Neurol. 2005;12:487–9. PubMed https://doi.org/10.1111/j.1468-1331.2005.00998.x

32. Papatsiros V, Vourvidis D, Tzitzis A, Meichanetsidis P, Stougiou D, Papaioannou D. *Streptococcus suis*: an important zoonotic pathogen for human prevention aspects. Vet World. 2011;216. https://doi.org/10.5455/vetworld.2011.216-221

33. Ágoston Z, Terhes G, Hannauer P, Gajdács M, Urbán E. Fatal case of bacteremia caused by *Streptococcus suis* in a splenectomized man and a review of the European literature. Acta Microbiol Immunol Hung. 2020;67:148–55. PubMed https://doi.org/10.1556/030.2020.01123

34. Gajdács M, Németh A, Knausz M, Barrak I, Stájer A, Mestyán G, et al. *Streptococcus suis*: An Underestimated Emerging Pathogen in Hungary? Microorganisms. 2020;8:1292. PubMed https://doi.org/10.3390/microorganisms8091292

35. Németh A, Knausz M, Schmidt P. *Streptococcus suis* okozta purulens meningitis különleges esete. Orv Hetil. 2019;160:30–4. PubMed https://doi.org/10.1556/650.2019.31243

36. Baddeley PG. *Streptococcus suis* infection. Occup Med (Lond). 1995;45:222. PubMed https://doi.org/10.1093/occmed/45.4.222-a

37. Camporese A, Tizianel G, Bruschetta G, Cruciatti B, Pomes A. Human meningitis caused by *Streptococcus suis*: the first case report from north-eastern Italy. Infez Med. 2007;15:111–4.

38. Mancini F, Adamo F, Creti R, Monaco M, Alfarone G, Pantosti A, et al. A fatal case of streptococcal toxic shock syndrome caused by *Streptococcus suis* carrying tet (40) and tet (O/W/32/O), Italy. J Infect Chemother. 2016;22:774–6. PubMed https://doi.org/10.1016/j.jiac.2016.05.011

39. Manzin A, Palmieri C, Serra C, Saddi B, Princivalli MS, Loi G, et al. *Streptococcus suis* meningitis without history of animal contact, Italy. Emerg Infect Dis. 2008;14:1946–8. PubMed https://doi.org/10.3201/eid1412.080679

40. Perseghin P, Bezzi G, Troupioti P, Gallina M. *Streptococcus suis* meningitis in an Italian blood donor. Lancet. 1995;346:1305–6. PubMed https://doi.org/10.1016/S0140-6736(95)91912-0

41. Parisi G, Spanu T, Mariani B, Rianda A, Tronci M. Meningitis caused by Streptococcus suis: a case report. Microbiol Med. 2010;25. 10.4081/mm.2010.2414 https://doi.org/10.4081/mm.2010.2414

42. Schultsz C, Jansen E, Keijzers W, Rothkamp A, Duim B, Wagenaar JA, et al. Differences in the population structure of invasive *Streptococcus suis* strains isolated from pigs and from humans in The Netherlands. PLoS One. 2012;7:e33854. PubMed https://doi.org/10.1371/journal.pone.0033854

43. de Ceuster LME, van Dillen JJ, Wever PC, Rozemeijer W, Louwerse ES. [*Streptococcus suis* meningitis in a meat factory employee]. Ned Tijdschr Geneeskd. 2012;156:A5080. PubMed

44. van de Beek D, Spanjaard L, de Gans J. *Streptococcus suis* meningitis in the Netherlands. J Infect. 2008;57:158–61. PubMed https://doi.org/10.1016/j.jinf.2008.04.009

45. van Samkar A, Brouwer MC, Schultsz C, van der Ende A, van de Beek D. *Streptococcus suis* meningitis in the Netherlands. J Infect. 2015;71:602–4. PubMed https://doi.org/10.1016/j.jinf.2015.07.001

46. van Jaarsveld BC, van Kregten E, van Kesteren RG, Rozenberg-Arska M, Bartelink AK. Fulminant sepsis caused by *Streptococcus suis* [in Dutch]. Ned Tijdschr Geneeskd. 1990;134:1462–4. PubMed

47. Zalas-Więcek P, Michalska A, Grąbczewska E, Olczak A, Pawłowska M, Gospodarek E. Human meningitis caused by *Streptococcus suis.* J Med Microbiol. 2013;62:483–5. PubMed https://doi.org/10.1099/jmm.0.046599-0

48. Seixas D, Lebre A, Crespo P, Ferreira E, Serra JE, Saraiva da Cunha JG. Meningite bacteriana aguda como doença ocupacional. Acta Med Port. 2014;27:519–21. PubMed https://doi.org/10.20344/amp.4735

49. Taipa R, Lopes V, Magalhães M. *Streptococcus suis* meningitis: first case report from Portugal. J Infect. 2008;56:482–3. PubMed https://doi.org/10.1016/j.jinf.2008.03.002

50. Pinto IV, Caixinha JP, Castro V, Marques A, Fonseca A, Vera J. Infecção por *Streptococcus suis*: uma causa rara de meningite no homem. Rev Port Doenc Infec. 2005;2:34–5.

51. Sena Esteves S, Carvalho de Almeida J, Abrunhosa J, Almeida E Sousa C, Arshad Q. *Pig's ear: Streptococcus suis* Meningitis and its associated inner ear implications. IDCases. 2017;10:55–7. PubMed https://doi.org/10.1016/j.idcr.2017.08.015

52. Dragojlović J, Milosević B, Sasić N, Pelemis M, Sasić M. *Streptococcus suis* infection—clinical manifestations. Med Pregl. 2005;58:236–9. PubMed https://doi.org/10.2298/MPNS0506236D

53. Alonso-Socas MM, Alemán-Valls R, Roldán-Delgado H, Gómez-Sirvent JL. Endocarditis y espondilodiscitis por *Streptococcus suis.* Enferm Infecc Microbiol Clin. 2006;24:354–5. PubMed https://doi.org/10.1157/13089675

54. Aspiroz C, Vela AI, Pascual MS, Aldea MJ. Endocarditis aguda por *Streptococcus suis* serogrupo 2 en España. Enferm Infecc Microbiol Clin. 2009;27:370–1. PubMed https://doi.org/10.1016/j.eimc.2008.11.010

55. Baena IM, Fernández C, Sánchez J, Calvente M, Aguadero V. *Streptococcus suis* type 2: emerging pathogen producer of meningitis [in Spanish]. Rev Esp Quimioter. 2012;25:293–4. PubMed

56. Geffner Sclarsky DE, Moreno Muñoz R, Campillo Alpera MS, Pardo Serrano FJ, Gómez Gómez A, Martínez-Lozano MD. Meningitis por *Streptococcus suis*. An Med Interna (Madrid). 2001 Jun;18(6) [cited 2022 Aug 24]. http://scielo.isciii.es/scielo.php?script=sci_arttext&pid=S0212-71992001000600007&lng=en&nrm=iso&tlng=en

57. Gómez-Zorrilla S, Ardanuy C, Lora-Tamayo J, Cámara J, García-Somoza D, Peña C, et al. *Streptococcus suis* infection and malignancy in man, Spain. Emerg Infect Dis. 2014;20:1067–8. PubMed https://doi.org/10.3201/eid2006.131167

58. Hidalgo A, Ropero F, Palacios R, García V, Santos J. Meningitis due to *Streptococcus suis* with no contact with pigs or porcine products. J Infect. 2007;55:478. PubMed https://doi.org/10.1016/j.jinf.2007.02.013

59. Riquelme E, Escribano E, Blanch JJ, Crespo MD. Meningitis aguda por *Streptococcus suis* en una carnicera. Enferm Infecc Microbiol Clin. 2008;26:256–7. PubMed https://doi.org/10.1016/S0213-005X(08)72700-9

60. López-Mestanza C, Bolaño-Navarro A, Sánchez-Sánchez A, Aldea-Mansilla C. Primer caso de shock séptico por *Streptococcus suis* de posible etiología alimentaria en España. Med Intensiva. 2016;40:516–8. PubMed https://doi.org/10.1016/j.medin.2016.01.006

61. Luengo-Alvarez J, Martín-Ruiz C, Sánchez Muñoz-Torrero JF, Iñiguez-Ovando R. Meningitis due to *Streptococcus suis*: a case report [in Spanish]. Enferm Infecc Microbiol Clin. 2006;24:352–4. PubMed https://doi.org/10.1157/13089674

62. Corrales-Arroyo MJ, Real-Francia MAD, Hernandez-Gonzalez A, Lopez-Gallardo G, Puebla JMM, Caston-Osorio JJ. Acute Meningitis by *Streptococcus suis.* Journal of Microbiology and Infectious Diseases. 2015;2:160–2. https://doi.org/10.5799/jmid.123130

63. Galbarro J, Franco-Álvarez de Luna F, Cano R, Angel Castaño M. Meningitis aguda y espondilodiscitis por *Streptococcus suis* en paciente sin contacto previo con cerdos o productos porcinos derivados. Enferm Infecc Microbiol Clin. 2009;27:425–7. PubMed https://doi.org/10.1016/j.eimc.2008.07.012

64. Martínez Avilés P, Jusdado Ruiz-Capillas JJ, Gómez Rodrigo J, Solís Villa J. [Sacroiliitis caused by *Streptococcus suis* type II]. An Med Interna. 1994;11:309. PubMed

65. Fernández-Ferro J, López-González FJ, Pardo F, Pías-Peleteiro JM. Meningitis aguda por *Streptococcus suis* en una criadora de cerdos. Enferm Infecc Microbiol Clin. 2011;29:396–7. PubMed https://doi.org/10.1016/j.eimc.2010.12.013

66. de la Hoz Adame ME, de la Rubia Martín F, Domínguez Fuentes B, García Gil D. Meningitis aguda por *Streptococcus suis*: a propósito de un caso en un paciente esplenectomizado. An Med Interna (Madrid). 2005 Oct;22(10) [cited 2022 Aug 24]. http://scielo.isciii.es/scielo.php?script=sci_arttext&pid=S0212-71992005001000020&lng=en&nrm=iso&tlng=en

67. Vela AI, Aspiroz C, Fortuño B, Tirado G, Sierra J, Martinez R, et al. Meningitis caused by an unusual genotype (ST3) of *Streptococcus suis.* Infection. 2013;41:701–3. PubMed https://doi.org/10.1007/s15010-012-0382-y

68. María Asensi J, Asensi V, Arias M, Moreno A, Pérez F, Navarro R. Meningitis por *Streptococcus suis*. A propósito de dos casos y revisión de la literatura. Enferm Infecc Microbiol Clin. 2001;19:186–8. https://doi.org/10.1016/S0213-005X(01)72606-7

69. Juncal AR, Pardo F, Rodríguez I, Pérez del Molino ML. [Meningitis by Streptococcus suis]. Enferm Infecc Microbiol Clin. 1997;15:120–1. PubMed

70. Fernández-Aguilar X, Gottschalk M, Aragon V, Càmara J, Ardanuy C, Velarde R, et al. Urban Wild Boars and Risk for Zoonotic *Streptococcus suis*, Spain. Emerg Infect Dis. 2018;24:1083–6. PubMed https://doi.org/10.3201/eid2406.171271

71. Díez de Los Ríos J, Reynaga E, García-Gonzàlez M, Càmara J, Ardanuy C, Cuquet J, et al. Clinical and Epidemiological Characteristics of *Streptococcus suis* Infections in Catalonia, Spain. Front Med (Lausanne). 2021;8:792233. PubMed https://doi.org/10.3389/fmed.2021.792233

72. Broullón-Dobarro A, Rey-Martínez M, Cabadas-Avión R. Meningitis aguda por *Streptococcus suis.* Rev Esp Anestesiol Reanim. 2014;61:223–4. PubMed https://doi.org/10.1016/j.redar.2013.06.004

73. Gustavsson C, Rasmussen M. Septic arthritis caused by *Streptococcus suis* serotype 5 in pig farmer. Emerg Infect Dis. 2014;20:489–90. PubMed https://doi.org/10.3201/eid2003.130535

74. Olearo F, Marinosci A, Stephan R, Cherkaoui A, Renzi G, Gaia N, et al. First case of *Streptococcus suis* infection in Switzerland: An emerging public health problem? Travel Med Infect Dis. 2020;36:101590. PubMed https://doi.org/10.1016/j.tmaid.2020.101590

75. Clarke D, Almeyda J, Ramsay I, Drabu YJ. Primary prevention of *Streptococcus suis* meningitis. Lancet. 1991;338:1147–8. PubMed https://doi.org/10.1016/0140-6736(91)92005-M

76. Meecham JS, Worth RC. Persistent diplopia following *streptococcus suis* type 2 meningitis. J R Soc Med. 1992;85:579–80. PubMed https://doi.org/10.1177/014107689208500927

77. Watkins EJ, Brooksby P, Schweiger MS, Enright SM. Septicaemia in a pig-farm worker. Lancet. 2001;357:38. PubMed https://doi.org/10.1016/S0140-6736(00)03570-4

78. Maher D. *Streptococcus suis* septicaemia presenting as severe acute gastro-enteritis. J Infect. 1991;22:303–4. PubMed https://doi.org/10.1016/S0163-4453(05)80022-2

79. Public Health England. Zoonoses Report UK 2017 [cited 2022 Aug 24]]. https://www.gov.uk/government/publications/zoonoses-uk-annual-reports

80. Schultsz C, van Dijk D, Wagenaar JA, van der Ende A. Zoonotic infections with *Streptococcus suis* in the Netherlands / IB 11-2013 | RIVM [cited 2022 Nov 8]. https://www.rivm.nl/weblog/zoonotische-infecties-met-streptococcus-suis-in-nederland-ib-11-2013

81. Main tables—Agriculture—Eurostat [cited 2023 Jan 26]. https://ec.europa.eu/eurostat/web/agriculture/data/main-tables

82. Population Censuses. Population Censuses. [cited 2023 Jan 23]. https://www.czso.cz/csu/czso/population-censuses

83. Current population. Federal Statistical Office. [cited 2023 Jan 23]. https://www.destatis.de/EN/Themes/Society-Environment/Population/Current-Population/_node.html

84. Demography—Population at the beginning of the month—France (including Mayotte since 2014) | Insee. [cited 2023 Jan 25]. https://www.insee.fr/en/statistiques/serie/001641607

85. Population. [cited 2023 Jan 23]. https://www.dst.dk/en/Statistik/emner/borgere/befolkning

86. Greece Population 1950–2023. [cited 2023 Jan 25]. https://www.macrotrends.net/countries/GRC/greece/population

87. Hungarian Central Statistical Office. [cited 2023 Jan 25]. https://www.ksh.hu/?lang=en

88. Estimated resident population—Years 2002–2019. [cited 2023 Jan 25]. http://dati.istat.it/Index.aspx?QueryId=12386&lang=en

89. INEbase. CONSULINE. [cited 2023 Jan 26].

https://www.ine.es/consul/serie.do?d=true&s=CP335&c=2&

90. StatLine—Population; key figures. [cited 2023 Jan 23].

https://opendata.cbs.nl/statline/#/CBS/en/dataset/37296eng/table?ts=1530690320120.

91. Population estimates—Office for National Statistics. [cited 2023 Jan 26].

https://www.ons.gov.uk/peoplepopulationandcommunity/populationandmigration/populationestimates#timeseries

92. Demography—Population—OECD Data. theOECD. [cited 2023 Jan 25].

http://data.oecd.org/pop/population.htm

93. Surveillance Atlas of Infectious Diseases [cited 2023 Jan 27].

https://www.ecdc.europa.eu/en/surveillance-atlas-infectious-diseases

94. Willemse N, Howell KJ, Weinert LA, Heuvelink A, Pannekoek Y, Wagenaar JA, et al. An emerging zoonotic clone in the Netherlands provides clues to virulence and zoonotic potential of *Streptococcus suis.* Sci Rep. 2016;6:28984. PubMed https://doi.org/10.1038/srep28984

95. Fillo S, Mancini F, Anselmo A, Fortunato A, Rezza G, Lista F, et al. Draft Genome Sequence of *Streptococcus suis* Strain SsRC-1, a Human Isolate from a Fatal Case of Toxic Shock Syndrome. Genome Announc. 2018;6:6. PubMed https://doi.org/10.1128/genomeA.00447-18

96. Olearo F, Marinosci A, Stephan R, Cherkaoui A, Renzi G, Gaia N, et al. First case of *Streptococcus suis* infection in Switzerland: An emerging public health problem? Travel Med Infect Dis. 2020;36:101590. PubMed https://doi.org/10.1016/j.tmaid.2020.101590

97. Werinder A, Aspán A, Söderlund R, Backhans A, Sjölund M, Guss B, et al. Whole-genome sequencing evaluation of MALDI-TOF MS as a species identification tool for *Streptococcus suis.* J Clin Microbiol. 2021;59:e0129721. PubMed https://doi.org/10.1128/JCM.01297-21

98. Chen C, Zhang W, Zheng H, Lan R, Wang H, Du P, et al. Minimum core genome sequence typing of bacterial pathogens: a unified approach for clinical and public health microbiology. J Clin Microbiol. 2013;51:2582–91. PubMed https://doi.org/10.1128/JCM.00535-13

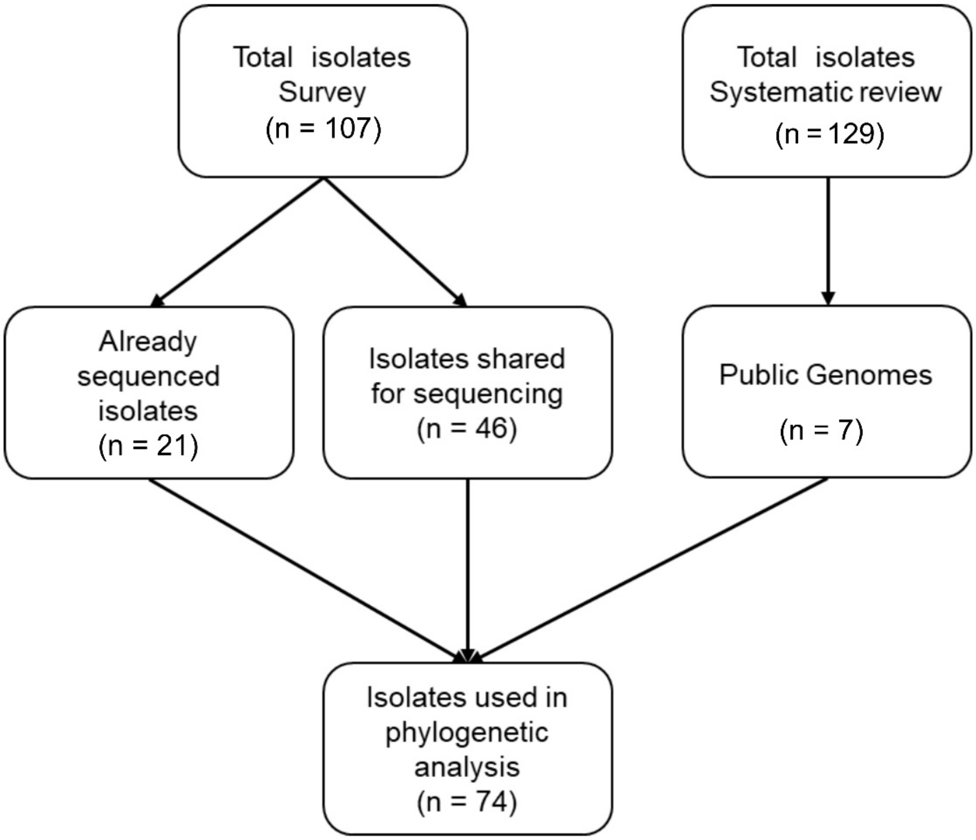

Appendix Figure 1. Human *Streptococcus suis* isolates included in phylogenic analysis.

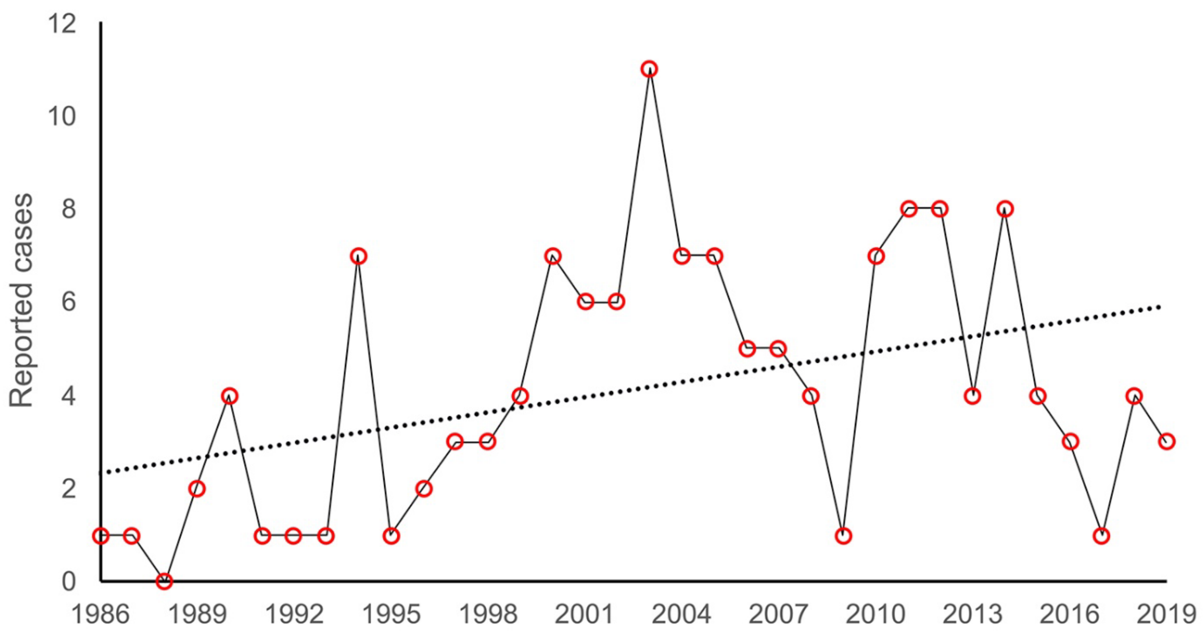

Appendix Figure 2. Reported human *Streptococcus suis* infections by year in Europe, combining survey and systematic review data.

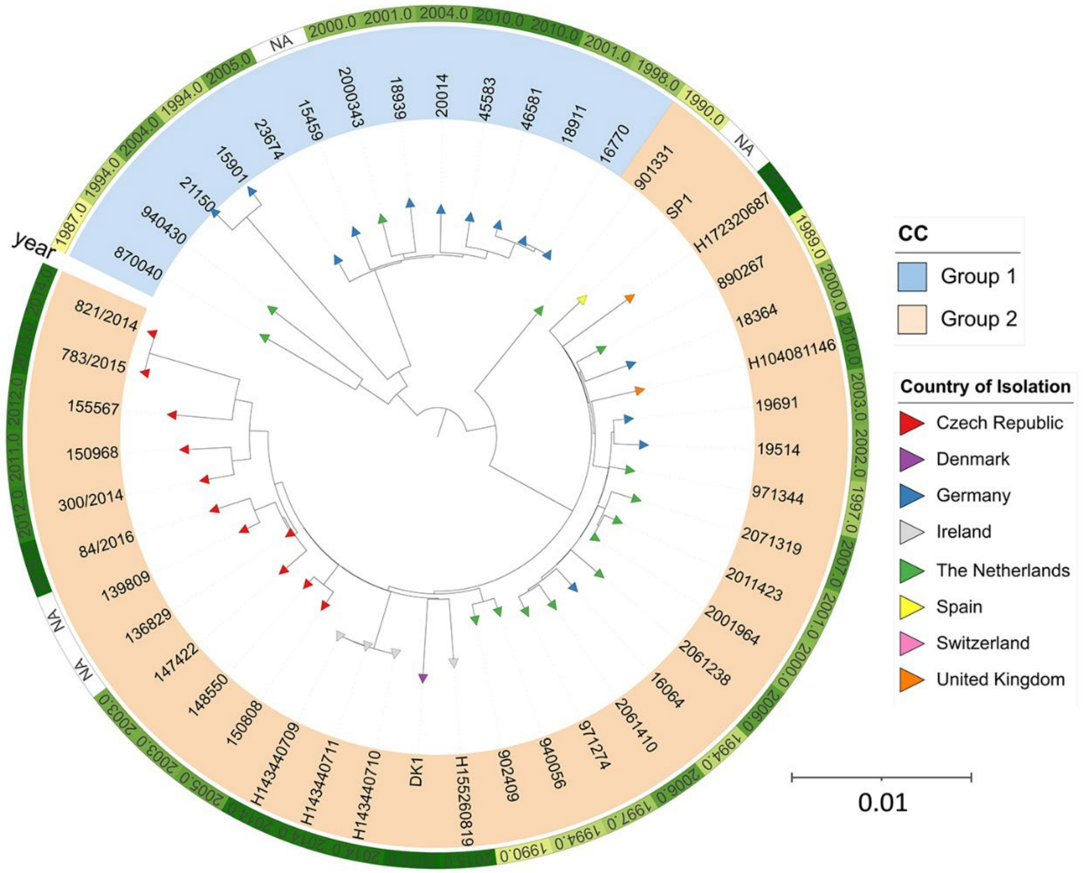

Appendix Figure 3. The 2 sublineages identified within zoonotic CC1 clade in Europe. Colored triangles indicate country of isolation. Colored ranges mark the 2 different groups: group 1 in blue and group 2 in orange. Outer ring indicates year of isolation; darker tones are associated with more recent and paler tones with more distant dates.
